# The serine protease prostasin regulates hepatic insulin sensitivity by modulating TLR4 signalling

**DOI:** 10.1038/ncomms4428

**Published:** 2014-03-11

**Authors:** Kohei Uchimura, Manabu Hayata, Teruhiko Mizumoto, Yoshikazu Miyasato, Yutaka Kakizoe, Jun Morinaga, Tomoaki Onoue, Rika Yamazoe, Miki Ueda, Masataka Adachi, Taku Miyoshi, Naoki Shiraishi, Wataru Ogawa, Kazuki Fukuda, Tatsuya Kondo, Takeshi Matsumura, Eiichi Araki, Kimio Tomita, Kenichiro Kitamura

**Affiliations:** 1 Department of Nephrology, Kumamoto University Graduate School of Medical Sciences, Kumamoto 860-8556, Japan; 2 Department of Medicine, Division of Diabetes and Endocrinology, Kobe University Graduate School of Medicine, Kobe 650-0017, Japan; 3 Department of Metabolic Medicine, Kumamoto University Graduate School of Medical Sciences, Kumamoto 860-8556, Japan; 4 These authors contributed equally to this work

## Abstract

The effects of high-fat diet (HFD) and postprandial endotoxemia on the development of type 2 diabetes are not fully understood. Here we show that the serine protease prostasin (PRSS8) regulates hepatic insulin sensitivity by modulating Toll-like receptor 4 (TLR4)-mediated signalling. HFD triggers the suppression of PRSS8 expression by inducing endoplasmic reticulum (ER) stress and increases the TLR4 level in the liver. PRSS8 releases the ectodomain of TLR4 by cleaving it, which results in a reduction in the full-length form and reduces the activation of TLR4. Liver-specific PRSS8 knockout (LKO) mice develop insulin resistance associated with the increase in hepatic TLR4. Restoration of PRSS8 expression in livers of HFD, LKO and *db/db* mice decreases the TLR4 level and ameliorates insulin resistance. These results identify a novel physiological role for PRSS8 in the liver and provide new insight into the development of diabetes resulting from HFD or metabolic endotoxemia.

## Abstract (web summary)

Hepatic insulin resistance is a hallmark of diabetes, but its aetiology is incompletely understood. Here, Uchimura and colleagues show that the serine protease prostasin cleaves Toll-like receptor 4 (TLR4) and regulates hepatic insulin sensitivity by modulating TLR4-mediated signalling.

## Graphical abstract

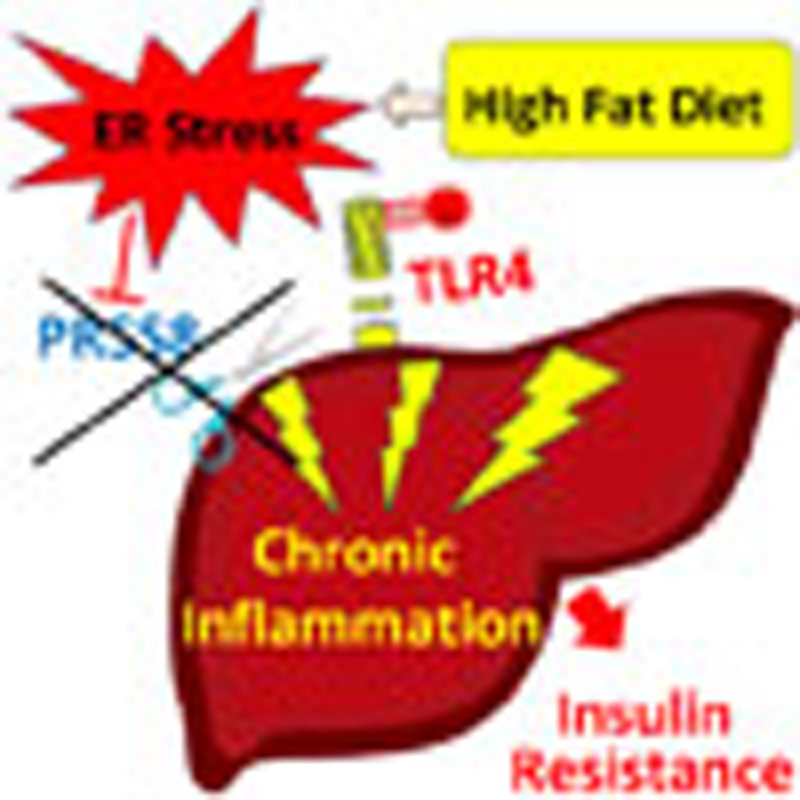

The epidemic surge in the prevalence of obesity-induced type 2 diabetes is one of the most serious worldwide health problems. Prolonged consumption of a high-fat diet (HFD) induces insulin resistance^1^,^2^,^3^,^4^,^5^,^6^,^7^, but the underlying molecular mechanisms are poorly understood.

A potential emerging mechanism involves the endoplasmic reticulum (ER), an organelle responsible for protein folding, maturation, quality control and trafficking. When the ER becomes stressed due to the accumulation of newly synthesized unfolded proteins, the unfolded protein response is activated^8^. Previous studies have demonstrated that mice engineered to have reduced ER capacity or increased levels of ER stress develop a higher degree of obesity when challenged with a HFD, and ER stress and activation of the unfolded protein response signalling pathway play a dominant role in the development of HFD-induced insulin resistance and type 2 diabetes^8^,^9^,^10^,^11^,^12^,^13^. However, the molecular mechanisms of the insulin resistance induced by ER stress remain unclear.

Toll-like receptors (TLRs) are pattern recognition receptors that initiate innate immune responses upon recognition of a wide range of pathogen-associated molecular patterns, including lipids, lipoproteins and proteins^14^. TLR4, a TLR family member, is activated by the lipidic domain of lipopolysaccharide (LPS), a gram-negative bacterial cell wall component^15^. Recent studies have shown that TLR4 signalling is important for HFD-induced insulin resistance, presumably by mediating inflammatory responses within adipose tissue, skeletal muscle, islets and the liver^16^,^17^,^18^,^19^,^20^. Collectively, these studies suggest the contribution of TLR4-mediated signalling to the development of type 2 diabetes.

ER stress and TLR4 signalling play dominant roles in the development of HFD-induced insulin resistance^21^. In this study, we demonstrate that the serine protease prostasin (PRSS8) protects the liver from chronic inflammation via the proteolytic cleavage and shedding of TLR4, consequently preventing the liver from developing insulin resistance. In addition, we found that HFD triggers the downregulation of PRSS8 via augmentation of ER stress in the liver, thereby contributing to the development of hepatic insulin resistance and diabetes. Our finding that PRSS8 controls the interaction between innate immunity and energy metabolism in the liver adds to the functional diversity of serine proteases in disease states and human health. Our study suggests a new pathogenic mechanism for HFD-induced insulin resistance and type 2 diabetes mellitus.

## Results

### HFD decreases hepatic PRSS8 expression by inducing ER stress

**Figure 1:**
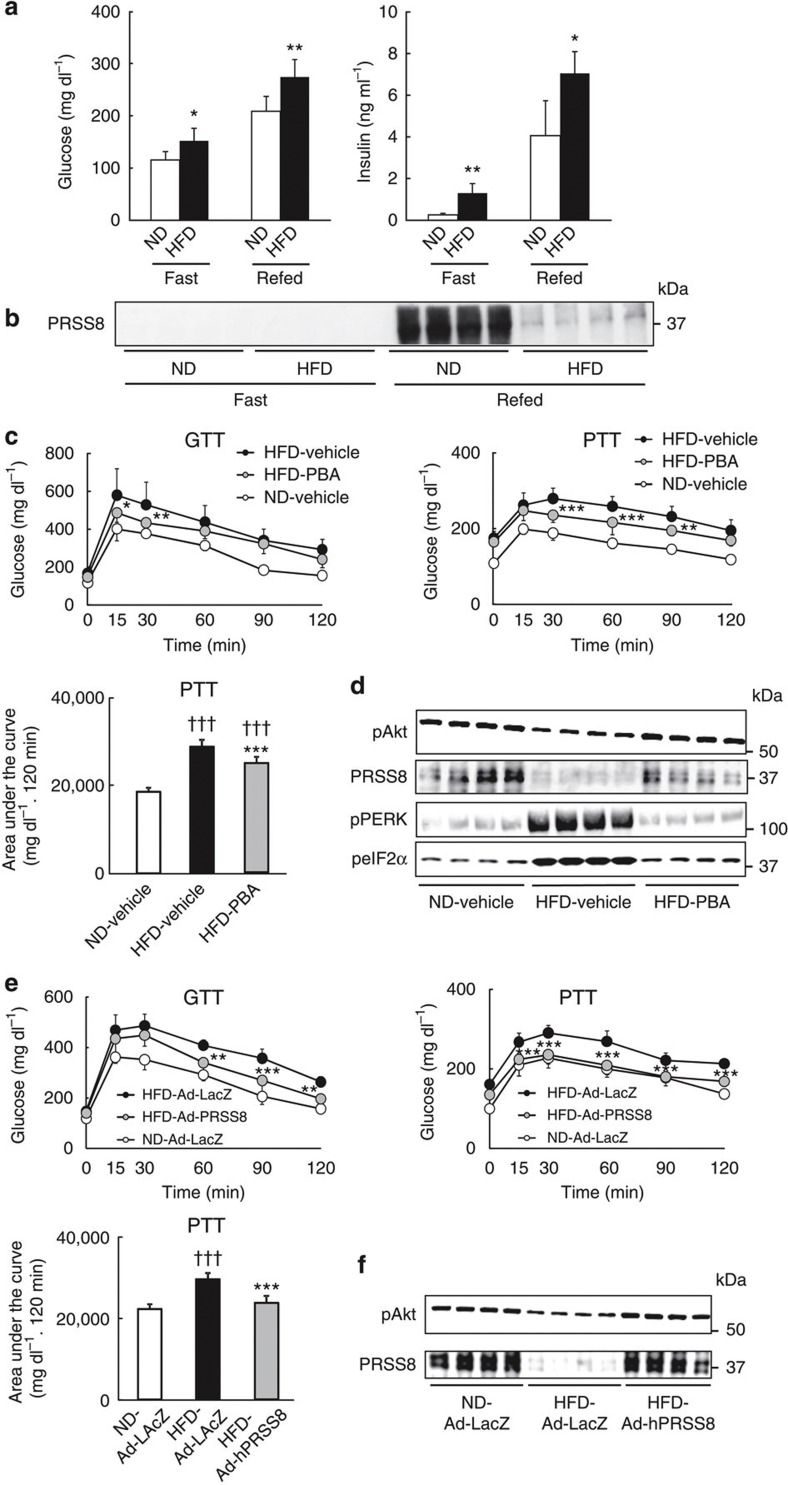
HFD-induced ER stress contributes to insulin resistance via a reduction in hepatic PRSS8. (**a**) Blood glucose and serum insulin levels under fasting and refeeding conditions in mice fed a ND or a HFD; (*n*=6–8 mice per group). Values are shown as the mean±s.d.; *P<0.05 and **P<0.01 versus ND (one-way analysis of variance (ANOVA)). (**b**) Western blotting analysis of PRSS8 in the livers of ND and HFD mice under fasting and refeeding conditions. A representative western blot image is shown (*n*=4 mice per group). (**c**) Blood glucose levels during GTT (2 g kg^−1^) or PTT (2 g kg^−1^) and the total area under the curve for the PTT in ND and HFD mice treated with vehicle or PBA (20 mM) (*n*=10 mice per group). Values are shown as the mean±s.d.; *P<0.05, **P<0.01 and ***P<0.001 for HFD–PBA versus HFD–vehicle (two-way ANOVA). ^†††^*P*<0.001 versus ND–vehicle (one-way ANOVA). (**d**) Western blotting for insulin-stimulated Akt phosphorylation and the level of PRSS8 and ER stress markers (pPERK and peIF-2α) in livers from ND and HFD mice treated with vehicle or PBA. Representative western blot images are shown (*n*=4 mice per group). (**e**) Blood glucose levels during a GTT or PTT and AUC for a PTT in ND and HFD mice infected with an adenovirus carrying LacZ (Ad-LacZ) or human PRSSS8 (Ad-hPRSS8) (*n*=10 mice per group). Values are shown as the mean±s.d.; **P<0.01 and ***P<0.001 for HFD-Ad-hPRSS8 versus HFD-Ad-LacZ (two-way ANOVA). ^†††^*P*<0.001 versus ND-Ad-LacZ (one-way ANOVA). (**f**) Western blotting for the insulin-stimulated phosphorylation of Akt and PRSS8 level in livers from ND and HFD mice infected with Ad-LacZ or Ad-hPRSS8. Representative western blot images are shown (*n*=4 mice per group).

While searching for molecules involved in the process of HFD-induced insulin resistance, we found that the expression of PRSS8, a glycosylphosphatidylinositol-anchored serine protease, was substantially reduced in HFD mouse livers compared with livers from mice fed a normal diet (ND) under refeeding conditions (Fig. 1a,b). This result indicates that HFD may trigger the downregulation of hepatic PRSS8. Because previous studies have demonstrated that ER stress plays a dominant role in the development of HFD-induced insulin resistance^3^,^9^,^11^,^12^,^21^,^22^, we hypothesized that ER stress reduces the expression of PRSS8 in the liver. Indeed, treatment with a chemical chaperone, 4-phenyl butyric acid (PBA), restored hepatic PRSS8 levels and improved insulin resistance, which was assessed by a glucose tolerance test (GTT) and pyruvate tolerance test (PTT), and insulin-stimulated Akt phosphorylation, via the amelioration of ER stress (Fig. 1c,d). Restoration of PRSS8 expression in HFD mouse livers with an adenovirus carrying human PRSS8 (Ad-hPRSS8) also reduced the blood glucose levels, which was measured by a GTT and PTT, and insulin-stimulated Akt phosphorylation in the liver as well (Fig 1e,f), suggesting a contribution of PRSS8 to glucose metabolism.

### LKO mice show glucose intolerance and insulin resistance

**Figure 2:**
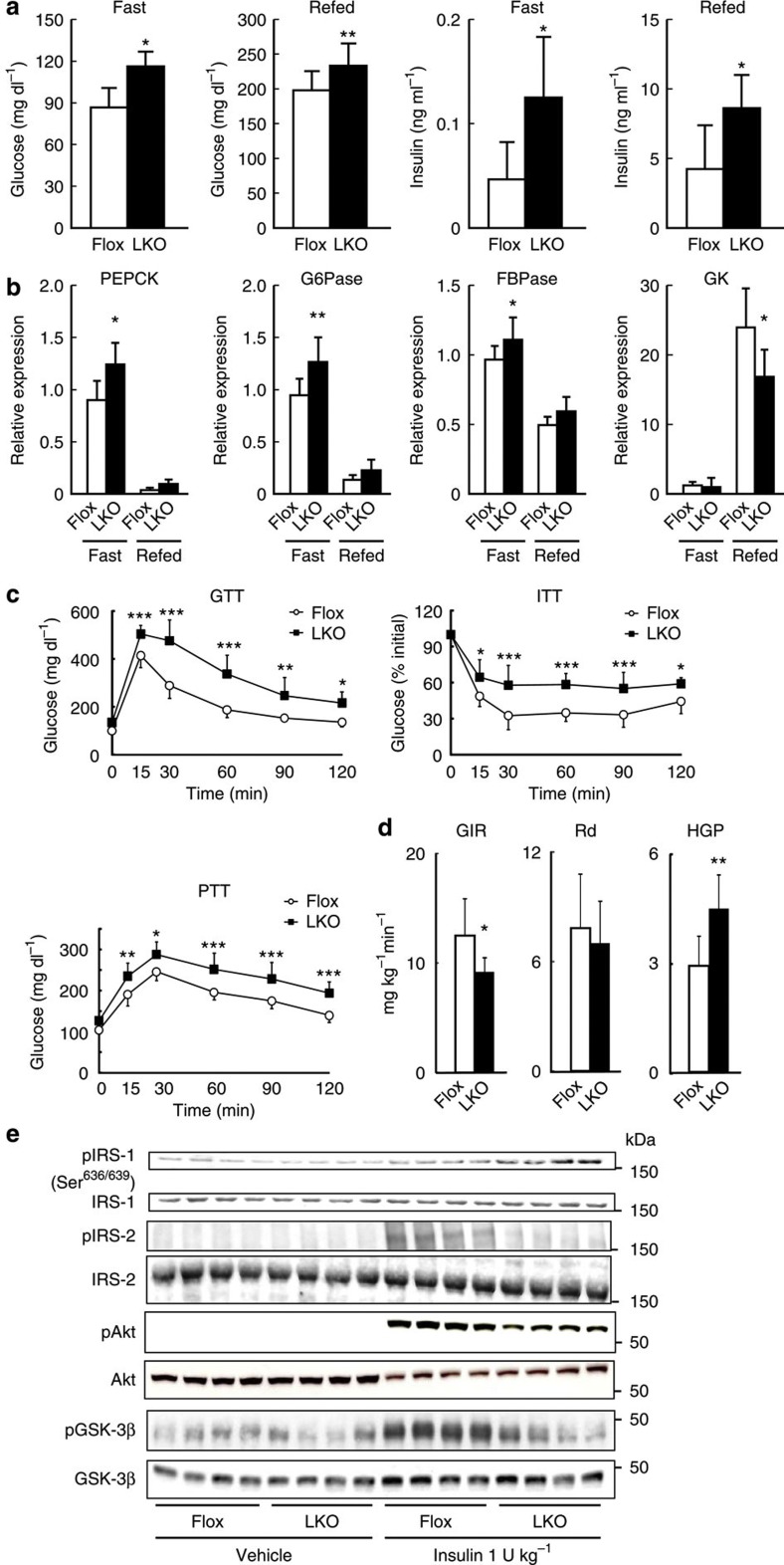
LKO mice have glucose intolerance and hepatic insulin resistance. (**a**) Blood glucose and serum insulin levels under fasting and refeeding conditions in Flox and LKO mice (*n*=10 mice per group). Values are shown as the mean±s.d.; *P<0.05 and **P<0.01 versus Flox (one-way ANOVA). (**b**) mRNA expression of PEPCK, G6Pase, FBPase and glucokinase (GK) in the liver under fasting and refeeding conditions (*n*=7–9 mice per group). Values are shown as the mean±s.d.; *P<0.05 and **P<0.01 versus Flox (one-way ANOVA). (**c**) Blood glucose levels during a GTT, (insulin 1.5 U kg^−1^) insulin tolerance test and PTT (*n*=10 mice per group). Values are shown as the mean±s.d.; *P<0.05, **P<0.01 and ***P<0.001 versus Flox (two-way ANOVA). (**d**) Glucose infusion rate, hepatic glucose production and glucose Rd in the hyperinsulinemic–euglycemic clamp study (*n*=5 mice per group). Values are shown as the mean±s.d.; *P<0.05 and **P<0.01 versus Flox (one-way ANOVA). (**e**) Western blotting for the insulin (1 U kg^−1^)-stimulated phosphorylation of IRS-1 (Ser^636/639^), IRS-2, Akt and GSK-3β in the liver. Representative western blot images are shown (*n*=4 mice per group).

To ensure that the reduction in hepatic PRSS8 is responsible for the insulin resistance phenotype, we generated liver-specific PRSS8 knockout (LKO) mice (Supplementary Fig. 1). Immunohistochemical analysis revealed a predominant localization of PRSS8 along the hepatic sinusoidal membrane that is distinct from CD10 staining (a marker for bile canaliculi localization) (Supplementary Fig. 2). The messenger RNA (mRNA) and protein levels of PRSS8 in livers from *PRSS8*^*lox/lox*^ mice (Flox) were significantly increased by refeeding after a 24-h fast, but hepatic PRSS8 mRNA and protein were completely absent in LKO mice (Supplementary Fig. 3). Although the body weight gain in LKO mice was similar to that in Flox mice (Supplementary Fig. 4), the LKO mice surprisingly demonstrated a significant elevation in blood glucose and serum insulin levels when compared with the Flox mice under fasting and refeeding conditions (Fig. 2a). No significant differences were observed in serum triglycerides, total cholesterol or free fatty acid levels (Supplementary Fig. 5). The increase in mRNA level of hepatic gluconeogenic enzymes (PEPCK, G6Pase and FBPase) due to fasting was significantly greater in LKO mice than Flox mice, and the increase in mRNA level of the glycolytic enzyme glucokinase upon refeeding was significantly lower in LKO mice than Flox mice (Fig. 2b). In the LKO mice, a GTT and insulin tolerance test demonstrated impaired glucose clearance and insulin sensitivity, and a PTT demonstrated increased hepatic gluconeogenesis (Fig. 2c). Hyperinsulinemic–euglycemic clamp studies demonstrated a reduced glucose infusion rate, an increase in hepatic glucose production, and an unchanged glucose disappearance rate (Rd) in LKO mice (Fig. 2d). Compared with Flox mice, LKO mice showed increased IRS-1 phosphorylation (Ser^639/639^) and decreased IRS-2, Akts and GSK-3β phosphorylation in the liver following insulin stimulation (Fig. 2e). The phosphorylation of Akt in skeletal muscle and adipose tissue was not significantly altered (Supplementary Fig. 6). No significant differences were observed in the expression of hepatic PRSS8, blood glucose levels during the GTT or PTT and insulin-stimulated Akt phosphorylation level between Flox and wild-type C57BL/6J mice (Supplementary Fig. 7). Because HFD markedly reduces the hepatic PRSS8 level in Flox mice, we could not find differences in insulin sensitivity between Flox and LKO mice (Supplementary Fig. 8).

### PRSS8 regulates the TLR4 level in the liver

**Figure 3:**
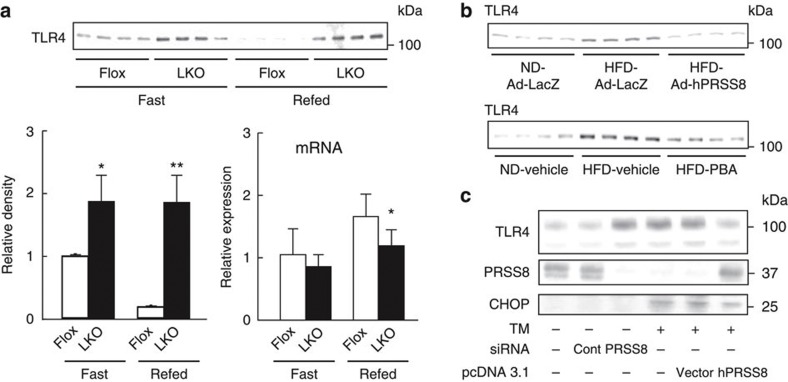
ER stress-mediated PRSS8 modulates hepatic TLR4 expression. (**a**) The expression of TLR4 was analysed by western blotting for protein levels and real-time PCR for mRNA levels in livers from Flox and LKO mice under fasting and refeeding conditions (*n*=4 mice per group). Values are shown as the mean±s.d.; *P<0.05 and **P<0.01 versus Flox (one-way ANOVA). (**b**) Protein level of TLR4 in livers from ND and HFD mice treated with Ad-LacZ or Ad-hPRSS8 (upper panel) and vehicle or PBA (lower panel) under fasting conditions were analysed by western blotting. Representative western blot images are shown (*n*=4 mice per group). (**c**) Western blotting for TLR4, PRSS8 and CHOP in HepG2 cells treated with and without tunicamycin in the presence of siRNA for Control or PRSS8 and pcDNA3.1 alone or pcDNA3.1-hPRSS8.

The contribution of TLR4-mediated signalling to the development of type 2 diabetes was recently demonstrated^2^,^17^,^18^,^19^,^20^,^21^,^23^,^24^,^25^,^26^,^27^,^28^. TLR4 activates proinflammatory kinases, such as JNK, IKK and p38, which directly impair insulin signalling via the serine phosphorylation of IRS-1. TLR4 activation also induces the expression of proinflammatory genes, resulting in an elevation of cytokines, chemokines, reactive oxygen species and eicosanoids to promote further insulin desensitization^17^,^18^,^20^. A protective role for PRSS8 against the inflammatory response to LPS was previously reported^29^, although the precise mechanism remains unclear. We hypothesized that PRSS8 modulates TLR4-mediated inflammatory signalling and consequently regulates hepatic insulin sensitivity. Disruption of hepatic PRSS8 resulted in a significant increase in the hepatic TLR4 level with a concomitant reduction in TLR4 mRNA, which suggests a negative feedback for mRNA expression through post-translational protein modification (Fig. 3a and Supplementary Fig. 9). However, LKO mice showed no significant change in the expression of other cell surface receptors regulating inflammation or glucose metabolism (Supplementary Fig. 10). Furthermore, the restoration of hepatic PRSS8 expression in HFD mice by Ad-hPRSS8 or treatment with PBA substantially decreased the hepatic TLR4 levels (Fig. 3b). Similarly, tunicamycin, an ER stress inducer, decreased PRSS8 expression with a concomitant increase in the TLR4 level in HepG2 cells, and PRSS8 overexpression reduced the tunicamycin-mediated elevation in TLR4 levels (Fig. 3c).

### LKO mice show an excessive response to LPS

**Figure 4:**
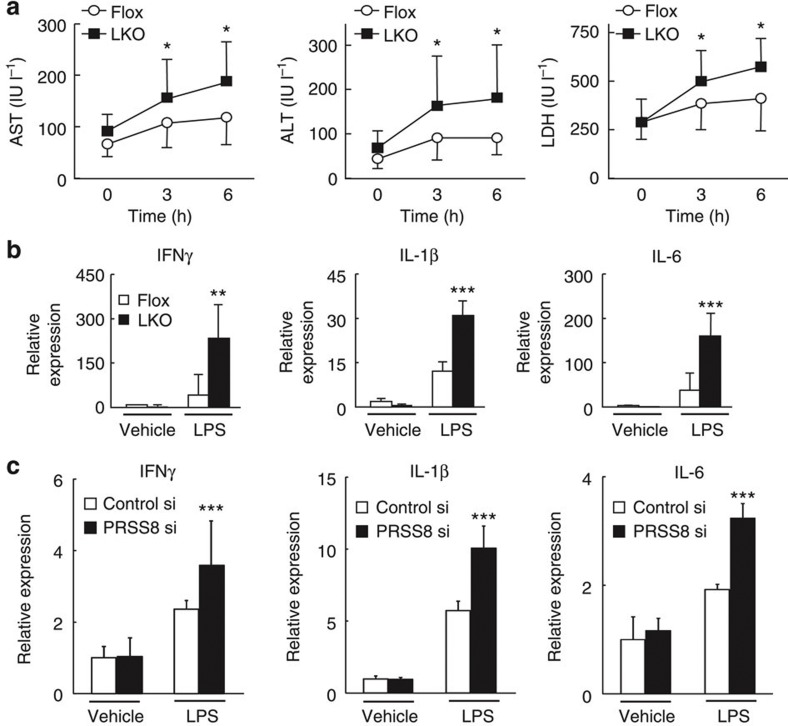
LKO mice and PRSS8-depleted HepG2 cells show an excessive response to LPS. (**a**) Time course of serum AST, ALT and LDH levels after intraperitoneal LPS injection (80 mg kg^−1^) (*n*=8 mice per group). *P<0.05 versus Flox (one-way ANOVA). Values are shown as the mean±s.d. (**b**) LPS-induced mRNA expression of proinflammatory cytokines in the livers of Flox and LKO mice 6 h after intraperitoneal injection (*n*=8 mice per group). **P<0.01 and ***P<0.001 versus Flox (one-way ANOVA). Values are shown as the mean±s.d. (**c**) HepG2 cells were transfected with Control siRNA or PRSS8 siRNA. The LPS-induced mRNA expression of proinflammatory cytokines was determined 12 h after treatment (*n*=6 per group). ***P<0.001 versus Control siRNA (one-way ANOVA). Values are shown as the mean±s.d.

**Figure 5:**
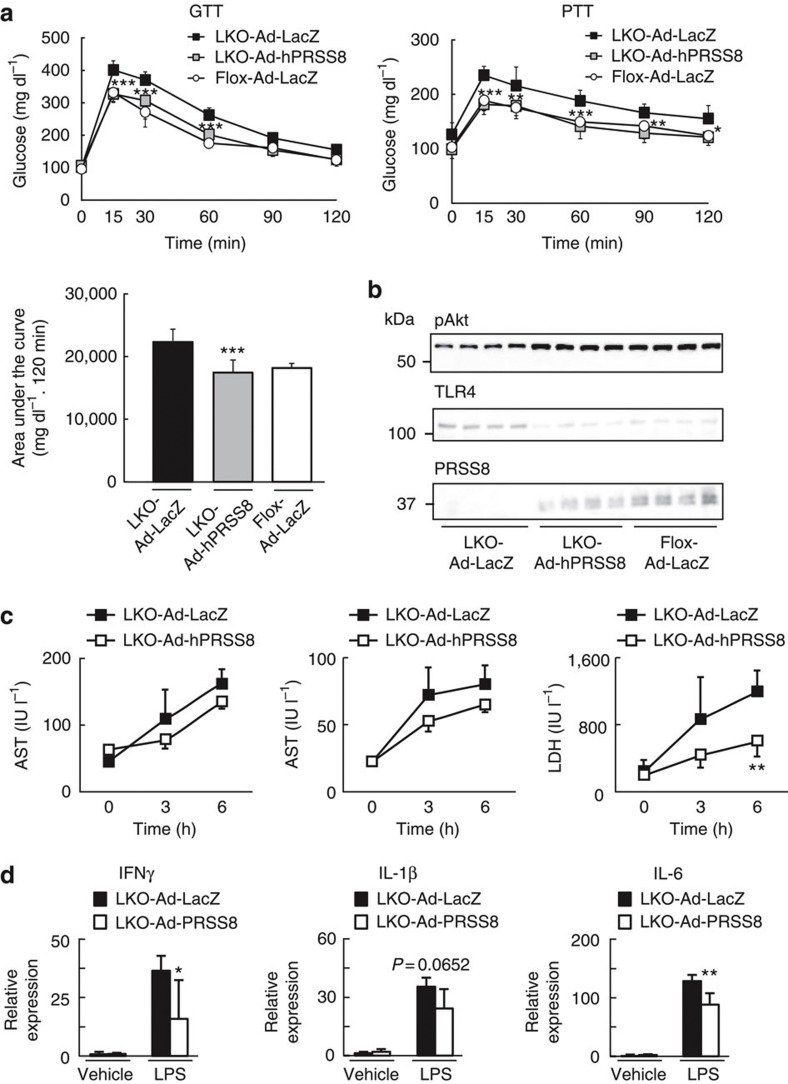
Restoration of hepatic PRSS8 expression ameliorates the insulin resistance and LPS-induced inflammatory response in LKO mice. (**a**) Blood glucose levels during a GTT and PTT and area under the curve for the PTT (*n*=10 mice per group). *P<0.05, **P<0.01 and ***P<0.001 for LKO-Ad-PRSS8 versus LKO-Ad-LacZ (two-way ANOVA). Values are shown as the mean±s.d. (**b**) Western blotting for the insulin-stimulated phosphorylation of Akt and level of TLR4 and PRSS8 in the liver (*n*=4 mice per group). (**c**) Serum AST, ALT and LDH levels after LPS (80 mg kg^−1^) injection in LKO mice receiving Ad-LacZ or Ad-hPRSS8 (*n*=8 mice per group). **P<0.01 versus LKO-Ad-LacZ (one-way ANOVA). Values are shown as the mean±s.d. (**d**) mRNA expression of proinflammatory cytokines 6 h after intraperitoneal LPS injection (*n*=8 mice per group). *P<0.05 and **P<0.01 versus LKO-Ad-LacZ (one-way ANOVA). Values are shown as the mean±s.d.

The intraperitoneal injection of LPS induced a substantial increase in the serum AST, ALT and LDH levels (Fig. 4a) and proinflammatory cytokines, such as IFNγ, IL-1β and IL-6, in livers from LKO mice compared with livers from Flox mice (Fig. 4b). Similarly, this prominent induction of proinflammatory cytokines by LPS was also observed in PRSS8-depleted HepG2 cells (Fig. 4c). These findings indicate that hepatocytes in LKO mice have an excessive inflammatory response to LPS that is at least partly mediated by the increased level of TLR4 in the liver. *In vivo* restoration of PRSS8 expression in the livers of LKO mice by Ad-hPRSS8 ameliorated the insulin resistance (Fig. 5a,b) and decreased the LPS-induced inflammatory responses (Fig. 5c,d).

### PRSS8 decreases TLR4 levels by the proteolytic shedding

**Figure 6:**
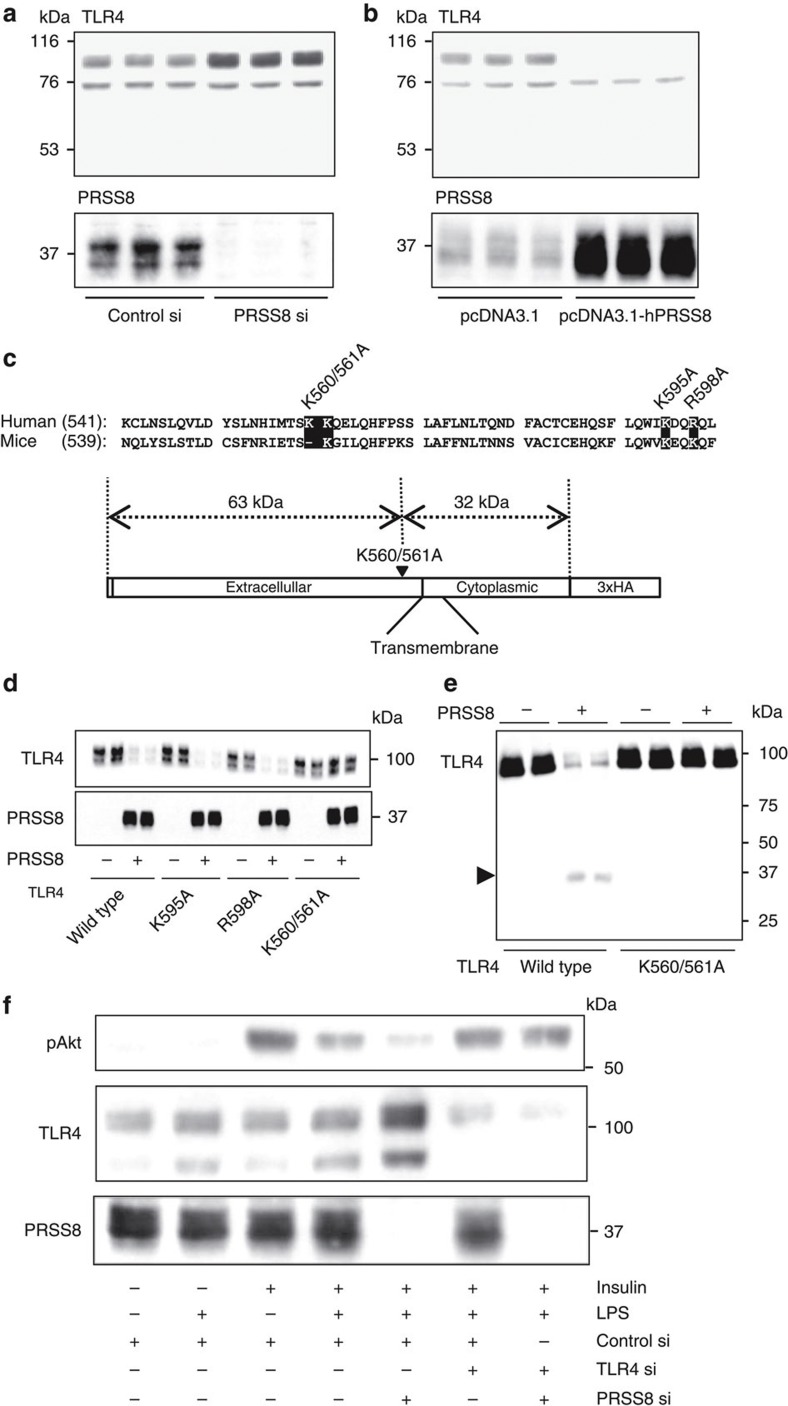
PRSS8 decreases the level of TLR4 at the plasma membrane via proteolytic shedding. (**a**) Western blotting for TLR4 and PRSS8 in HepG2 cells transfected with Control siRNA and PRSS8 siRNA. (**b**) Western blotting for TLR4 and PRSS8 in HepG2 cells transfected with pcDNA3.1 or pcDNA3.1-hPRSS8. (**c**) Lys and Arg residues conserved between humans and mice are highlighted in black with white lettering. These residues are located within the extracellular domain and are ~30 kDa upstream from the carboxy terminus. Site-directed mutagenesis was conducted by replacing these residues with Ala (K560A/K561A, K595A or R598A). (**d**) Wild-type or mutant human TLR4 was transfected with or without PRSS8 into HEK293 cells, and the level of TLR4 and PRSS8 was determined by western blotting. (**e**) Immunoprecipitated wild-type or K560/561A mutant HA-tagged TLR4 was incubated with or without recombinant human PRSS8 and subjected to immunoblotting using an anti-HA antibody. (**f**) HepG2 cells transfected with Control, PRSS8 or TLR4 siRNA or PRSS8 and TLR4 siRNA were pretreated with LPS (50 μg ml^−1^) and then insulin (500 nM)-stimulated Akt phosphorylation was determined by western blotting.

Because PRSS8 is anchored to the plasma membrane outside the cell^30^, we hypothesized that PRSS8 reduces the level of TLR4 on the plasma membrane via proteolytic cleavage and shedding of the TLR4 extracellular domain. An small interfering RNA (siRNA)-mediated decrease in PRSS8 expression increased the level of TLR4 in HepG2 cells (Fig. 6a). Conversely, PRSS8 overexpression markedly decreased the level of TLR4 (Fig. 6b). In addition, we detected a fragment that was 30 kDa smaller than full-length TLR4 using an antibody directed against the ectodomain of TLR4 in the culture media of HepG2 cells overexpressing PRSS8 (Supplementary Fig. 11), suggesting that PRSS8 engages in proteolytic processing of TLR4 at the cell surface. Mutational analysis of TLR4 cleavage by PRSS8 was conducted by replacing the Lys or Arg residues in TLR4, which are amino acids targeted by PRSS8 (ref. 31)^31^ located ~30 kDa upstream from the carboxy terminus of human TLR4 (K560A/K561A, K595A and R598A) in the ectodomain, with Ala residues (Fig. 6c). The level of the wild-type protein and K595A and R598A mutants was decreased when these constructs were co-transfected with human PRSS8 in HEK293 cells, whereas the level of the K560A/K561A mutant was unchanged (Fig. 6d). We examined the proteolytic processing of TLR4 by PRSS8 *in vitro*. HA-tagged wild-type or K560A/K561A-mutant TLR4 was immunoprecipitated, incubated with recombinant human PRSS8 and subjected to immunoblotting with an anti-HA antibody. Treatment of wild-type TLR4 with PRSS8 resulted in a loss of the full-length form, which was accompanied by the appearance of the cleaved 30 kDa form. The K560A/K561A mutant was completely protected against cleavage by PRSS8 (Fig. 6e). These findings indicate that PRSS8 releases the ectodomain of TLR4 by proteolytic shedding and consequently attenuates inflammatory signalling through TLR4. A reduction in PRSS8 expression by siRNA in HepG2 cells provoked a significant decrease in phosphorylated Akt following insulin stimulation in the presence of the TLR4 ligand LPS. Furthermore, double knockdown of TLR4 and PRSS8 ameliorated the suppression of Akt phosphorylation observed in the PRSS8-depleted cells following insulin and LPS stimulation (Fig. 6f). The TLR2 ligand zymosan did not affect the insulin-stimulated Akt phosphorylation in PRSS8-depleted HepG2 cells, suggesting that PRSS8 specifically regulates the TLR4 signalling pathway (Supplementary Fig. 12).

### Reduction in hepatic TLR4 improves insulin resistance

**Figure 7:**
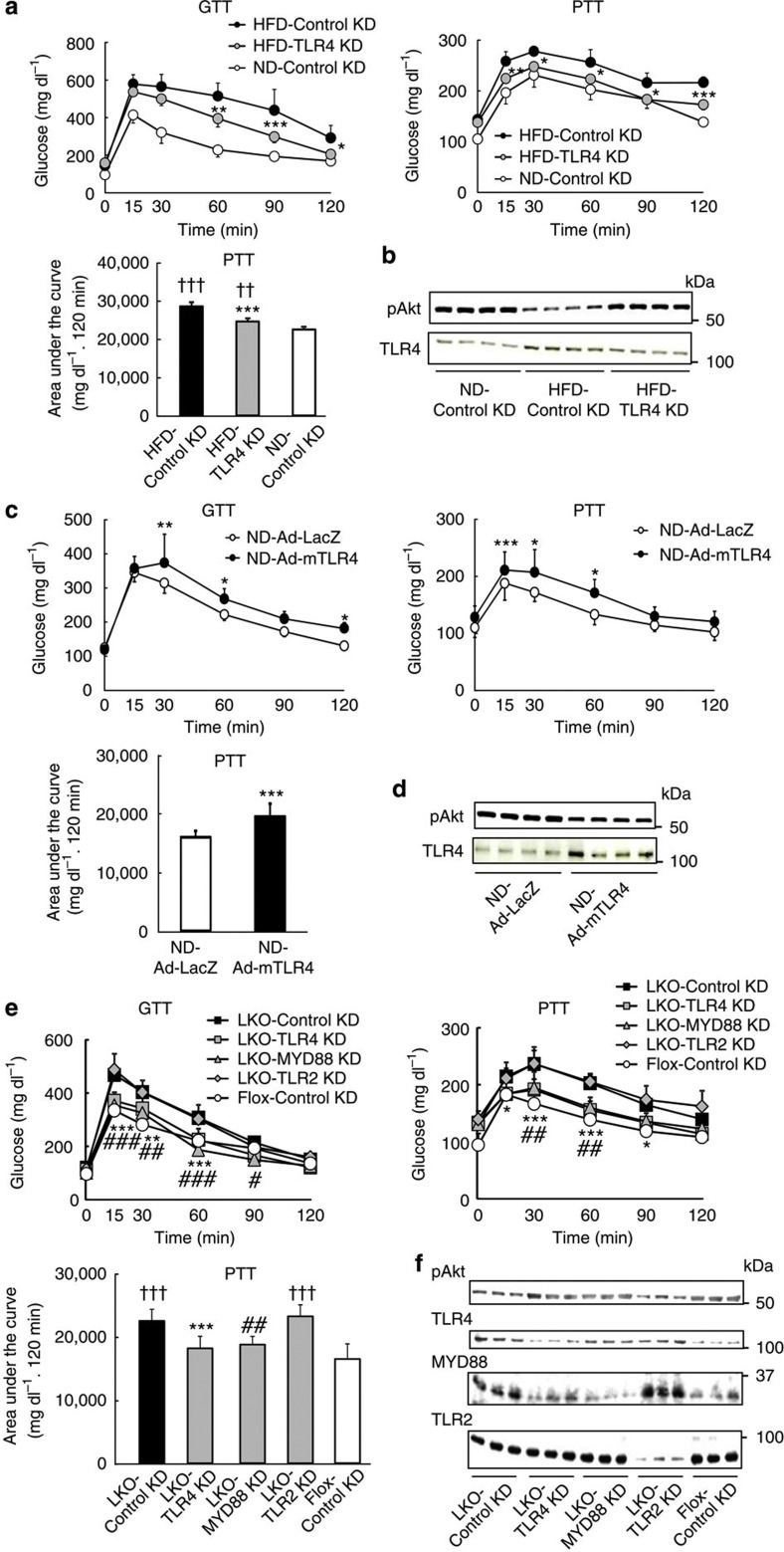
Reduction in hepatic TLR4 improves insulin resistance in HFD and LKO mice. (**a**) Blood glucose levels during a GTT and PTT, and the AUC for the PTT in ND and HFD mice transfected with Control siRNA or TLR4 siRNA (*n*=8 mice per group). Values shown are the mean±s.d.; *P<0.05, **P<0.01 and ***P<0.001 for HFD-TLR4 KD versus HFD-Control KD (two-way ANOVA). ^††^*P*<0.01 and ^†††^*P*<0.001 versus ND-Control KD (one-way ANOVA). (**b**) Western blotting for the insulin-stimulated Akt phosphorylation and TLR4 protein levels in livers from ND and HFD mice transfected with Control siRNA and TLR4 siRNA. Representative western blot images are shown (*n*=4 mice per group). (**c**) Blood glucose levels during a GTT and PTT, and the AUC for the PTT in ND mice infected with an adenovirus carrying LacZ (Ad-LacZ) or mouse TLR4 (Ad-mTLR4) (*n*=8 mice per group). Values are shown as the mean±s.d.; *P<0.05, **P<0.01 and ***P<0.001 versus ND-Ad-LacZ (two-way ANOVA). (**d**) Western blotting for the insulin-stimulated Akt phosphorylation and TLR4 protein levels in livers from ND mice infected with Ad-LacZ or Ad-mTLR4. Representative western blot images are shown (*n*=4 mice per group). (**e**) Blood glucose levels during a GTT and PTT, and the area under the curve for the PTT in LKO mice transfected with Control, TLR4, MYD88 or TLR2 siRNA (*n*=6–8 mice per group). Values are shown as the mean±s.d. *P<0.05, **P<0.01 and ***P<0.001 for LKO-TLR4 KD versus LKO-Control KD (two-way ANOVA). ^#^*P*<0.05, ^##^*P*<0.01 and ^###^*P*<0.001 for LKO-MYD88 KD versus LKO-Control KD (two-way ANOVA). ^†††^*P*<0.001 versus Flox-Control KD (one-way ANOVA). (**f**) Western blotting for insulin-stimulated Akt phosphorylation and the TLR4, MYD88 and TLR2 protein level in livers from LKO mice transfected with Control, TLR4, MYD88 and TLR2 siRNA. Representative western blot images are shown (*n*=3 mice per group).

Knockdown of TLR4 by siRNA in HFD mouse livers significantly improved their insulin resistance (Fig. 7a,b). Conversely, overexpression of TLR4 by adenovirus in the livers of wild-type mice induced insulin resistance even under a ND (Fig. 7c,d, and Supplementary Fig. 13). The siRNA-mediated reduction in TLR4, but not TLR2, in livers of LKO mice (Supplementary Figs 14, 15 and 16) significantly ameliorated the insulin resistance (Fig. 7e,f). Knockdown of myeloid differentiation primary response gene 88 (MYD88) (Supplementary Fig. 17), an adaptor protein for TLR4, improved the insulin resistance in LKO mice (Fig. 7e,f), and overexpression of PRSS8 in the liver did not decrease blood glucose levels during the GTT and PTT in systemic TLR4 KO mice (Supplementary Fig. 18). These findings indicate the significance of hepatic TLR4 signalling in the development of insulin resistance. Saberi *et al.*^17^ demonstrated an important role for hematopoietic TLR4 in the obesity-induced insulin resistance in the liver and adipose tissue following a 16-week treatment with a HFD. Our HFD mice fed for only 2 weeks, and LKO mice exhibited insulin resistance even with a ND at 8 weeks of age. These findings suggest the possibility that hepatic TLR4 is more involved in physiological glucose metabolism, and hematopoietic TLR4 is critical in disease states such as obesity-induced type 2 diabetes.

### Hepatic PRSS8 mediates insulin resistance in *db/db* mice

**Figure 8:**
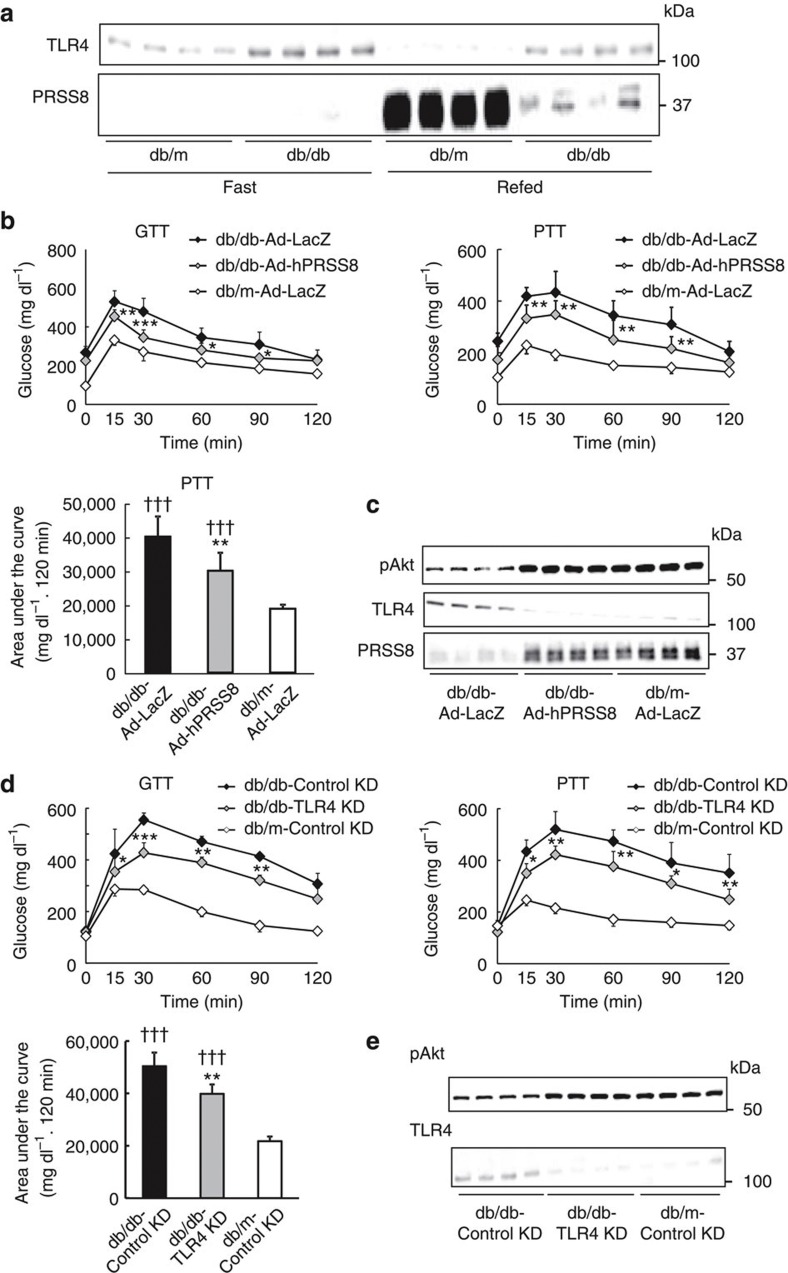
Restoration of hepatic PRSS8 levels improves insulin resistance in *db/db* mice. (**a**) The levels of PRSS8 and TLR4 in livers from *db/m* and *db/db* mice under fasting and refeeding conditions were analysed by western blotting. Representative western blot images are shown (*n*=4 mice per group). (**b**) Blood glucose levels during a GTT and PTT, and the AUC for the PTT in *db/m* and *db/db* mice infected with Ad-LacZ or Ad-hPRSS8 (*n*=10 mice per group). Values are shown as the mean±s.d.; *P<0.05, **P<0.01 and ***P<0.001 for *db/db*-Ad-hPRSS8 versus *db/db*-Ad-LacZ (two-way ANOVA). ^†††^*P*<0.001 versus *db*/m-Ad-LacZ (one-way ANOVA). (**c**) Western blotting for insulin-stimulated Akt phosphorylation and the level of TLR4 and PRSS8 in livers from *db/m* and *db/db* mice infected with Ad-LacZ or Ad-hPRSS8. Representative western blot images are shown (*n*=4 mice per group). (**d**) Blood glucose levels during a GTT and PTT, and the AUC for the PTT in *db/m* and *db/db* mice transfected with Control or TLR4 siRNA (*n*=8 mice per group). Values are shown as the mean±s.d.; *P<0.05, **P<0.01, and ***P<0.001 for *db/db*-TLR4 KD versus *db/db*-Control KD (two-way ANOVA). ^†††^*P*<0.001 versus *db/m*-Control KD (one-way ANOVA). (**e**) Western blotting for insulin-stimulated Akt phosphorylation and the TLR4 protein level in livers from *db/m* and *db/db* mice transfected with Control or TLR4 siRNA. Representative western blot images are shown (*n*=4 mice per group).

To further validate our hypothesis, we next determined the hepatic PRSS8 level in genetically diabetic *db/db* mice. The mRNA and protein levels of PRSS8 were substantially reduced, and the protein levels of TLR4 was reciprocally increased in the livers of *db/db* mice when compared with control *db/m* mice under refeeding conditions (Fig. 8a and Supplementary Fig. 19). The concomitant suppression of TLR4 mRNA in *db/db* livers implicates a post-translational mechanism as observed in Fig. 3a (Supplementary Fig. 19). Forced expression of human PRSS8 or TLR4 depletion in *db/db* mouse livers ameliorated the insulin resistance (Fig. 8b–e).

### Serum PRSS8 levels are correlated with BMI and HOMA-IR

**Figure 9:**
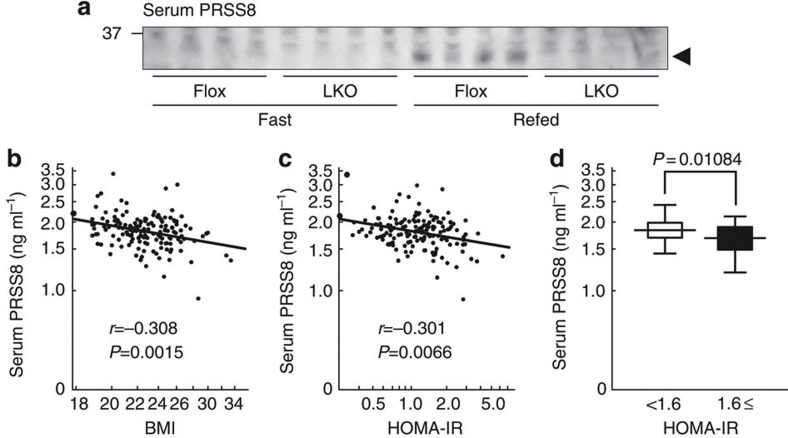
The serum PRSS8 levels are negatively correlated with BMI and HOMA-IR in healthy human subjects (*n*=153). (**a**) Western blotting analysis of serum PRSS8. A representative western blot image is shown (*n*=4 mice per group). (**b**) Correlation between serum PRSS8 levels and BMI. (**c**) Correlation between serum PRSS8 levels and HOMA-IR. (**d**) Comparison of the serum PRSS8 level in healthy human subjects with a HOMA-IR <1.6 (*n*=111) and a HOMA-IR ≥1.6 (*n*=42). Statistical comparisons were made with the Mann–Whitney *U*-test. Values are shown as the mean±s.d.

Because the soluble form of PRSS8 can be detected in serum, we examined the effects of the liver-specific disruption of PRSS8 on the serum PRSS8 levels. Although serum PRSS8 was increased in refed Flox mice when compared with fasted Flox mice, it was not detectable in LKO mice (Fig. 9a), which suggests that a major component of serum PRSS8 may originate from the liver, and serum PRSS8 levels may serve as markers for hepatic PRSS8 levels. We investigated the correlation between serum PRSS8 levels and body mass index (BMI) or homoeostasis model assessment-insulin resistance (HOMA-IR) values in healthy human subjects. The serum PRSS8 levels were negatively correlated with BMI and HOMA-IR (Fig. 9b,c). Individuals with a HOMA-IR ≥1.6 had slightly but significantly decreased serum PRSS8 levels when compared with those with a HOMA-IR <1.6 (Fig. 9d).

## Discussion

The dietary lipid-mediated facilitation of LPS incorporation into chylomicrons and the phagocytosis of gram-negative bacteria by gut enterocytes contribute to postprandial endotoxemia^5^,^27^,^32^. In patients with type 2 diabetes, elevated plasma LPS levels are associated with insulin resistance^33^. Thus, chronically elevated circulating gut-generated LPS or ‘metabolic endotoxemia’ could result in sustained systemic inflammatory signalling through TLR4 (refs 34, 35). Our results demonstrate a novel role for hepatic PRSS8 in the regulation of insulin sensitivity via the proteolytic shedding of TLR4 in the liver. The reduction in hepatic PRSS8 mediated by HFD-induced ER stress results in the elevation of hepatic TLR4 and exposes the liver to the circulating TLR4 ligands, such as LPS and dietary saturated fatty acids, supplied by the portal vein, which consequently lead to the development of hepatic insulin resistance.

## Methods

### Generation of PRSS8 knockout mice

The experimental outline for generating the *PRSS8*^*lox*^ and *PRSS8*^*Δ*^ alleles is provided in Supplementary Fig. 1. Albumin promoter-driven Cre transgenic mice (*Alb-Cre* mice) were purchased from The Jackson Laboratory (Bar Harbor, ME, USA). Mice with a floxed *PRSS8* allele (*PRSS8*^*lox/lox*^) were intercrossed with *Alb-Cre* mice to generate *AlbCrePRSS8*^*lox/+*^ mice. *AlbCrePRSS8*^*lox/+*^ mice were crossed with *PRSS8*^*lox/lox*^ mice to obtain *AlbCrePRSS8*^*lox/lox*^ (LKO) mice. *PRSS8*^*lox/lox*^(Flox) mice were used as controls in this study. Genotyping was performed by PCR amplification of the tail DNA from each mouse at 4 weeks of age. The PCR primers for Cre recombinase were 5′-GCGGTCTGGCAGTAAAAACTATC-3′ and 5′-GTGAAACAGCATTGCTGTCACTT-3′. The primers for the floxed *PRSS8* alleles were 5′-CTGTAGCTGCCTGTACAACATTA-3′ and 5′-CAGGAAGCATAGGTAGAAGTCAGAG-3′. All of the mouse lines were maintained in a background derived from C57BL/6J.

### Animal studies

Mice were housed under a 12 h light–dark cycle and provided normal chow. All experiments in this study were conducted using male littermates between 8 and 12 weeks of age unless otherwise stated. Mice were fed a ND or HFD (60% fat) for 2 weeks starting at 5 weeks of age. For the PBA studies, mice were administered PBA in drinking water (20 mM). Liver-specific *PRSS8*^−/−^ mice and *db/db* mice received a ND and water *ad libitum*. For the fasting and refeeding experiments, mice were deprived of food for 16 h (fasting) followed by a high-sucrose diet for 16 h (refeeding). Hepatic insulin signals were measured based on the phosphorylation of IRS-1 (Ser^636/639^), IRS-2, Akt and GSK-3β following a 5 min treatment with intravenous insulin (1 U kg^−1^). LPS-induced liver injury was evaluated according to the serum AST, ALT and LDH levels as determined by commercial kits. The mRNA expression of proinflammatory cytokines, including IFNγ, IL-1β and IL-6, was determined 6 h after the intraperitoneal injection of LPS (80 mg kg^−1^).

All animal experiments were approved by Animal Care committee of Kumamoto University and were performed in accordance with institutional guidelines.

### *In vivo* siRNA transfections

LKO mice were injected with 10 mg kg^−1^ TLR4 (*Silencer* Select Validated siRNA, ID: s75207, Ambion), MYD88 (ID: s70236), TLR2 (ID: s76898) or control siRNA (ID: 4459405) using Invivofectamine2.0 (Invitrogen). Two days after tail vein injection, the GTT, PTT and insulin signals were determined.

### Adenovirus injections

The adenovirus vectors (Ad-hPRSS8, Ad-mTLR4 and Ad-LacZ) in which the expression of human prostasin, mice TLR4 and LacZ cDNA were under the control of the cytomegalovirus promoter/enhancer were constructed and prepared using the Transpose-Ad system (Qbiogene, Illkirch, France). All viruses were amplified in transcomplemental 293 cells and purified by caesium chloride gradient ultracentrifugation. Mice were injected with 1 × 10^10^ p.f.u. adenovirus via the tail vein. Five days after the injection, a GTT and PTT were performed, and insulin and LPS signals were determined.

### Metabolic tests

The glucose, insulin and PTTs were performed with mice fasted overnight. Mice received an intraperitoneal injection of glucose (2 g kg^−1^), insulin (1.5 U kg^−1^) or pyruvate (2 g kg^−1^), and blood glucose was assayed immediately before and after the injection at specified times.

### Hyperinsulinemic–euglycemic clamp studies

At least 2 days before experiments, the mice were anesthetized with pentobarbital sodium (50 mg kg^−1^), and an indwelling catheter was inserted into the right internal jugular vein. After overnight fasting, [3-^3^H] glucose (0.02 μCi min^−1^, GE Healthcare) was infused for 2 h (basal period) before the initiation of the clamp studies to estimate the rate of basal glucose turnover. A blood sample was collected at the end of the basal period. A 120 min hyperinsulinemic–euglycemic clamp study was conducted with the prime-continuous infusion of human insulin (2.5 mU kg^−1^ min^−1^) and [3-^3^H] glucose (0.1 μCi min^−1^). Blood glucose was monitored every 10 min and maintained at 90–110 mg dl^−1^ administering 40% glucose. Blood was sampled via tail tip bleeds at 90, 100, 110 and 120 min to determine the glucose Rd. The Rd was calculated according to non-steady-state equations, and hepatic glucose production was calculated as the difference between the Rd and the exogenous glucose infusion rate.

### Real-time PCR

TaqMan probes for PEPCK (ID: Mm00440637), G6Pase (ID: Mm00839363), FBPase (ID: Mm00490181), GK (ID: Mm00439129), TLR4 (ID: Mm00445273), IFNγ (ID: Mm01168134 and Hs00989291), IL-1β (ID: Mm01336189 and Hs01555410), IL-6 (ID: Mm00446190 and Hs00985639) and PRSS8 (ID: Mm00504792) were purchased from Applied Biosystems. Total RNA extracted from tissues or cells was reverse transcribed into cDNA with oligo(dT) primers using Superscript III. Real-time PCR was performed with the ABI PRISM 7900 Sequence Detector System (Applied Biosystems). Statistical analysis of the results was performed with the ^Δ^*C*_t_ value (*C*_t gene of interest_–*C*_t 18S or Actin_). Relative gene expression was obtained using the ^ΔΔ^*C*_t_ method (*C*_t sample_–*C*_t calibrator_).

### Immunoblotting and Immunoprecipitation

Tissue or cell lysates were prepared by homogenization in lysis buffer (25 mM HEPES, 10 mM Na_4_P_2_O_7_·10 H_2_O, 100 mM NaF, 5 mM EDTA, 2 mM Na_3_VO_4_, 1% Triton X-100 and 1 mM PMSF). Protein lysates were subjected to SDS–polyacrylamide gel electrophoresis and probed with primary antibodies directed against PRSS8 (ref. 36)^36^, IRS-1 (1:1,000, Upstate), phospho-IRS-1 (Ser^636/639^), Akt, phospho-Akt, GSK-3β, phospho-GSK-3β, phospho-NF-κB, MYD88 (1:1,000, Cell Signaling), human TLR4 (1:1,000, R&D Systems), mouse TLR4 and TLR2 (1:1,000, Santa Cruz Biotechnology). For IRS-2 immunoprecipitation, 1 mg of liver extracts was incubated with IRS-2 antibody (1:50, EMD Millipore) for 16 h at 4 °C. Protein G-Sepharose was then added followed by an additional 2-h incubation at 4 °C. After washing three times with lysis buffer, the immunocomplexes were resolved by SDS–polyacrylamide gel electrophoresis and probed with anti-phosphotyrosine antibody (1:1,000, Upstate).

### Cell culture and transfection

HepG2 and HEK293 cells were purchased from ATCC and maintained in Dulbecco’s modified Eagle medium containing 10% fetal bovine serum. For gene-silencing experiments, HepG2 cells were transfected with human PRSS8 siRNA (*Silencer* Select Validated siRNA, ID: s11274, Ambion), human TLR4 siRNA (ID: s14194) or control siRNA (ID: 4390844) using Lipofectamine RNAiMAX (Invitrogen) according to the manufacturer’s instructions. For heterologous expression experiments, cDNA for human PRSS8 (accession code NM-002773, NCBI Reference Sequence Database) was isolated from a human kidney cDNA library (Clontech) by PCR and subcloned into the pcDNA3.1 vector (Invitrogen). cDNA for human MD-2 (pUNO1-hMD2a) and HA-tagged human TLR4 (HA-TLR4) (pUNO-hTLR04a-HA3x) was purchased from InvivoGen. HEK293 cells were transfected with human PRSS8, human MD-2 or human HA-TLR4 using Lipofectamine (Invitrogen) according to the manufacturer’s protocol.

### *In vitro* studies

HepG2 cells were transfected with human PRSS8 siRNA, human TLR4 siRNA, human PRSS8 cDNA or HA-tagged human TLR4 cDNA. Cells were harvested 72 h after transfection, and the levels of PRSS8 and TLR4 were determined by immunoblotting. Insulin signalling in HepG2 cells was determined by the phosphorylation of Akt following a 15-min insulin treatment (500 nM) in the presence or absence of 24 h pretreatment with LPS (50 μg ml^−1^) or zymosan (100 μg ml^−1^). For tunicamycin studies, cells were treated with 5 μg ml^−1^ tunicamycin for 24 h under various experimental conditions.

### *In vitro* proteolytic analysis

Site-directed mutagenesis was performed using the QuikChange Lightning Site-Directed Mutagenesis Kit (Agilent Technologies). Oligonucleotides were designed to replace Lys or Arg residues with Ala at positions Lys^595^, Arg^598^, Lys^560^, and Lys^561^ in the human HA-TLR4 cDNA. Wild-type or mutant HA-TLR4 cDNA together with MD-2 cDNA (5 μg each) was transfected into HEK293 cells (10 cm dish), and the cell lysates were immunoprecipitated with 10 μg of anti-HA antibody (MBL). The immunoprecipitates were then incubated with 500 ng of recombinant human PRSS8 (ref. 37)^37^ in the reaction buffer (25 mM Tris, pH 9.0, 1% CHAPS) for 30 min at 37 °C followed by immunoblotting with an anti-HA antibody (InvivoGen).

### Measurement of serum PRSS8 levels

Healthy male subjects (mean [±s.d.] age: 46.1±11.3 years, *n*=153) who visited Yatsushiro General Hospital (Yatsushiro, Japan) for annual health examinations were recruited to participate in this study. This study was approved by the Institutional Review Board of Kumamoto University (No. 540). Written informed consent was obtained from each subject, and serum PRSS8 levels were determined using the RIA method developed in our laboratory^38^. In this RIA system, recombinant human PRSS8 was used as a reference standard. RIA was performed using a double-antibody method. PRSS8 antiserum diluted at 1:1,000 exhibited the ability to specifically bind 22% of added radioligands in the absence of unlabelled recombinant PRSS8 when 100 μl of diluted antiserum and 100 μl of labelled prostasin peptide (10,000 c.p.m.) were added to each assay tube containing 300 μl of 1% BSA–PBS. The radioactivity in each precipitate was measured in a gamma counter (Aloka). Fasting serum glucose and insulin levels were measured using commercial kits, and the BMI was evaluated. The relationship between serum PRSS8 level and BMI or HOMA-IR was examined using nonparametric Spearman’s rank correlation coefficient with StatFlex ver. 6 (Artech). The serum PRSS8 levels in healthy male subjects with a HOMA-IR <1.6 and a HOMA-IR ≥1.6 were compared using the Mann–Whitney *U*-test.

## Author contributions

K.U., M.H., T.M. and K.K. conceptualized and designed the research; K.U., M.H., T.M., Y.M., Y.K., J.M., T.O., R.Y., M.U. W.O. and K.F. performed the experiments; K.U., M.H., T.M., R.Y., K.F. and T.M. analysed the data; K.U., T.K., T.M., E.A., K.T. and K.K. interpreted the results of the experiments; K.U., M.H., T.M. and Y.M. prepared the figures; K.U., M.H. and K.K. drafted the manuscript; K.U. T.K., T.M., E.A., K.T. and K.K. edited and revised the manuscript; and K.U., H.M., T.M., Y.K., J.M., T.O., R.Y., M.U., M.A., T.M., N.S., W.O., K.F., T.K., T.M., E.A., K.T. and K.K. approved the final version of the manuscript.

## Additional information

**How to cite this article**: Uchimura, K. *et al.* The serine protease prostasin regulates hepatic insulin sensitivity by modulating TLR4 signalling. *Nat. Commun.* 5:3428 doi: 10.1038/ncomms4428 (2014).

## Supplementary Material

Supplementary Information Supplementary Figures 1-19
