# Supplementary material for: The serine protease prostasin regulates hepatic insulin sensitivity by modulating TLR4 signalling
Source: Nat Commun. 2014 Mar 11;5:3428. doi: 10.1038/ncomms4428 (PMC3959208; doi:10.1038/ncomms4428)
Supplement: Supplementary Information — Supplementary Figures 1-19 [file ncomms4428-s1.pdf]

Supplementary Figure 1.

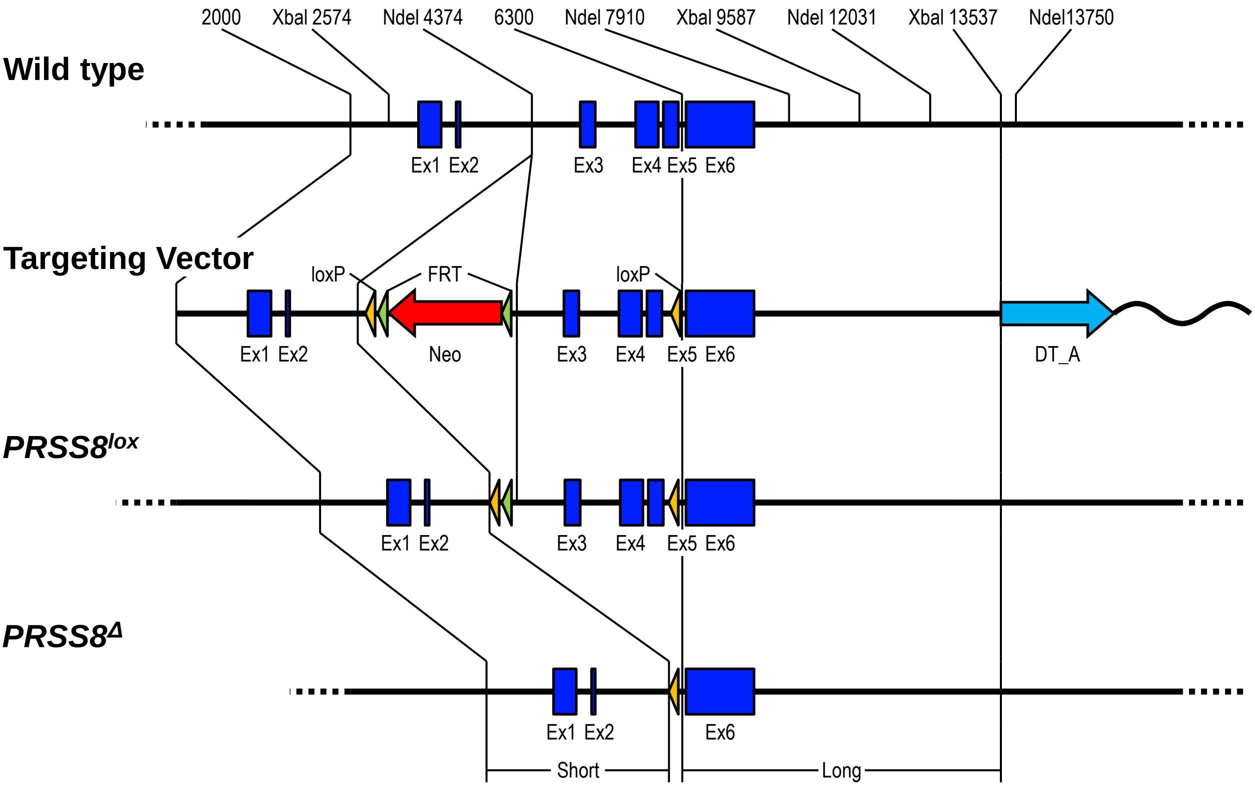

Supplementary Figure 1. *PRSS8^{lox}* allele used for Cre-mediated *PRSS8* gene inactivation. Schematic diagram (not to scale) for the generation of the *PRSS8^{lox}* allele. We generated a targeting vector consisting of a 2.4 kb 5' homologous sequence followed by a *Pgk*-neo cassette that is flanked by two Frt sites. The targeted 2.0 kb region consists of exons 3-5 flanked by two *loxP* sites followed by a 7.2 kb 3' homologous sequence. Deletion of exons 3-5 of the *PRSS8* gene leads to a frameshift within exon 6 that results in a stop codon and the generation of a protein truncated at the C-terminus. The targeting vector was electroporated into C57BL/6J embryonic stem cells. Colonies were isolated following selection with G418 and analyzed by southern blotting. Following injection into BALB/c donor blastocysts, male chimeras were obtained that transmitted the targeted *PRSS8^{neolox}* allele through the germ line. We deleted the neo cassette by Flp-mediated recombination *in vivo* using chicken β-actin promoter-driven Flp transgenic mice. The resulting offspring were genotyped by PCR amplification of DNA collected from the tail. Viable heterozygous *PRSS8$^{lox/+}$* and homozygous mutant *PRSS8$^{lox/lox}$* mice were obtained. Cre-mediated loxP recombination produced a *PRSS8$^{\Delta}$* allele.

Supplementary Figure 2.

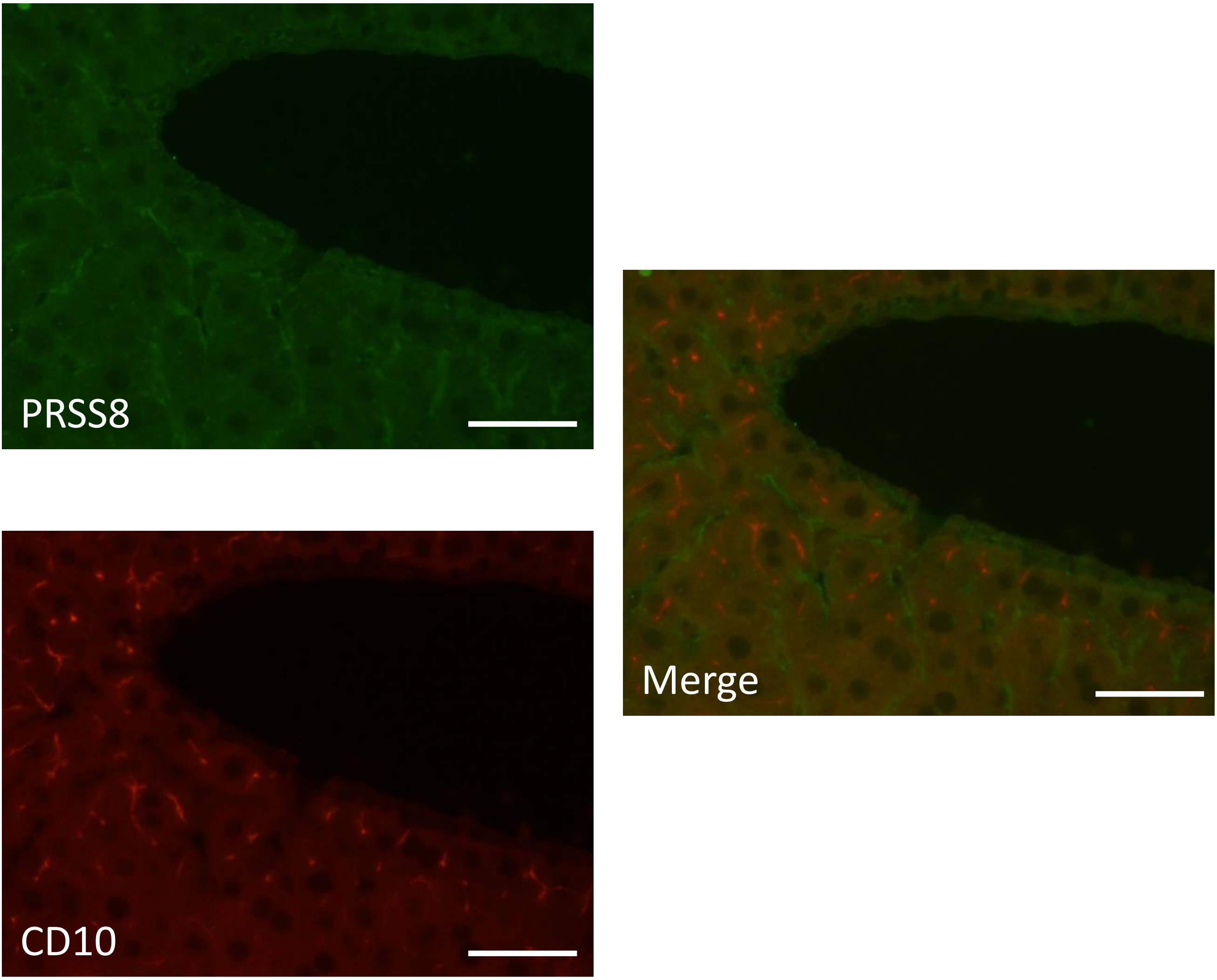

Supplementary Figure 2. Immunohistochemical analysis of PRSS8 (green) and CD10 (red) in the livers from Flox mice.

Immunostaining was performed on 5-μm paraffin sections. Sections were incubated with anti-PRSS8 (Proteintech) and anti-CD10 (Abcam) antibodies, and visualized with Alexa 488 and 555. Scale bars, 40 μm.

Supplementary Figure 3.

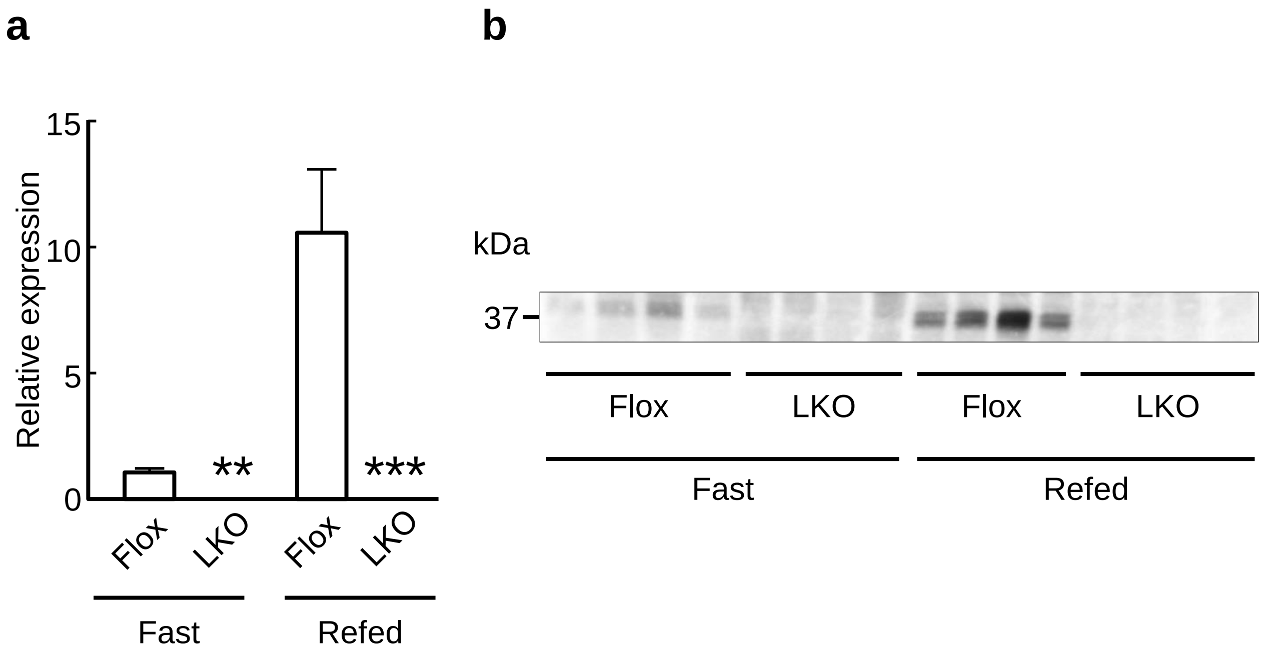

Supplementary Figure 3. Expression of PRSS8 in the livers from Flox and LKO mice.
(a) mRNA levels of PRSS8 in the livers from Flox and LKO mice under fasting or refeeding conditions (n=8 mice per group). ***p*<0.01 and ****p*<0.001 vs. Flox (one-way ANOVA). Values are shown as the mean ± s.d. **(b)** protein levels of PRSS8 in the livers from Flox and LKO mice under fasting or refeeding conditions. Representative western blot image was shown (n=8 mice per group).

Supplementary Figure 4.

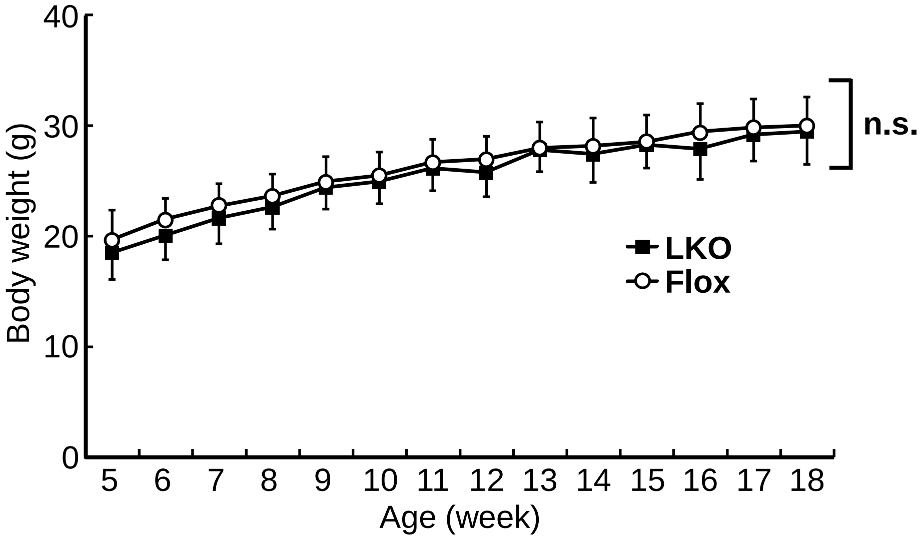

Supplementary Figure 4. The body weight gain in LKO mice was similar to that in Flox mice. Body weight changes in Flox and LKO mice fed a normal diet (n=25-28). Statistical comparisons were made by one-way ANOVA. Values are shown as the mean ± s.d.

Supplementary Figure 5.

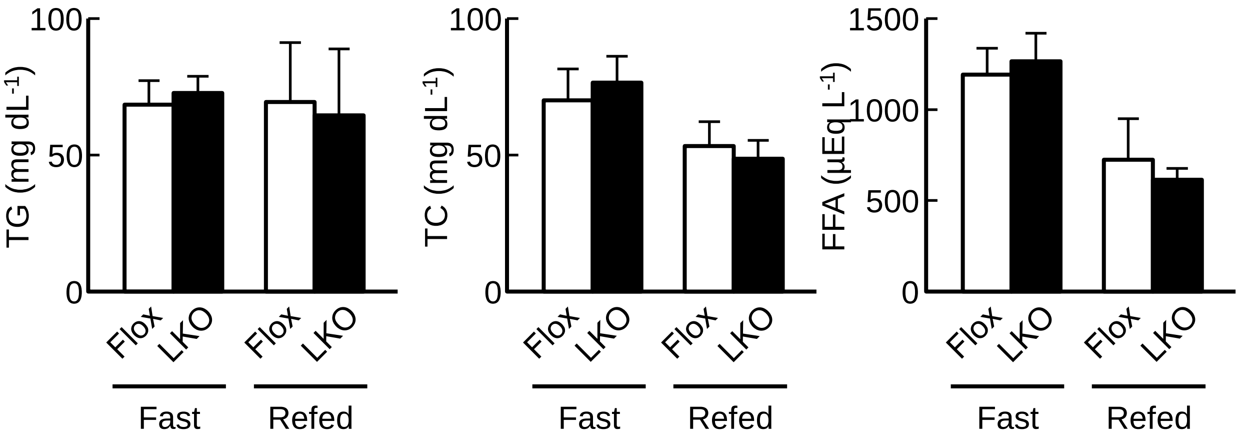

Supplementary Figure 5. Serum metabolic parameters in Flox and LKO mice. Serum levels of triglycerides (TG), total cholesterol (TC), and free fatty acids (FFA) in Flox and LKO mice under fasting or refeeding conditions (n=6-8 mice per group). Values are shown as the mean ± s.d.

Supplementary Figure 6.

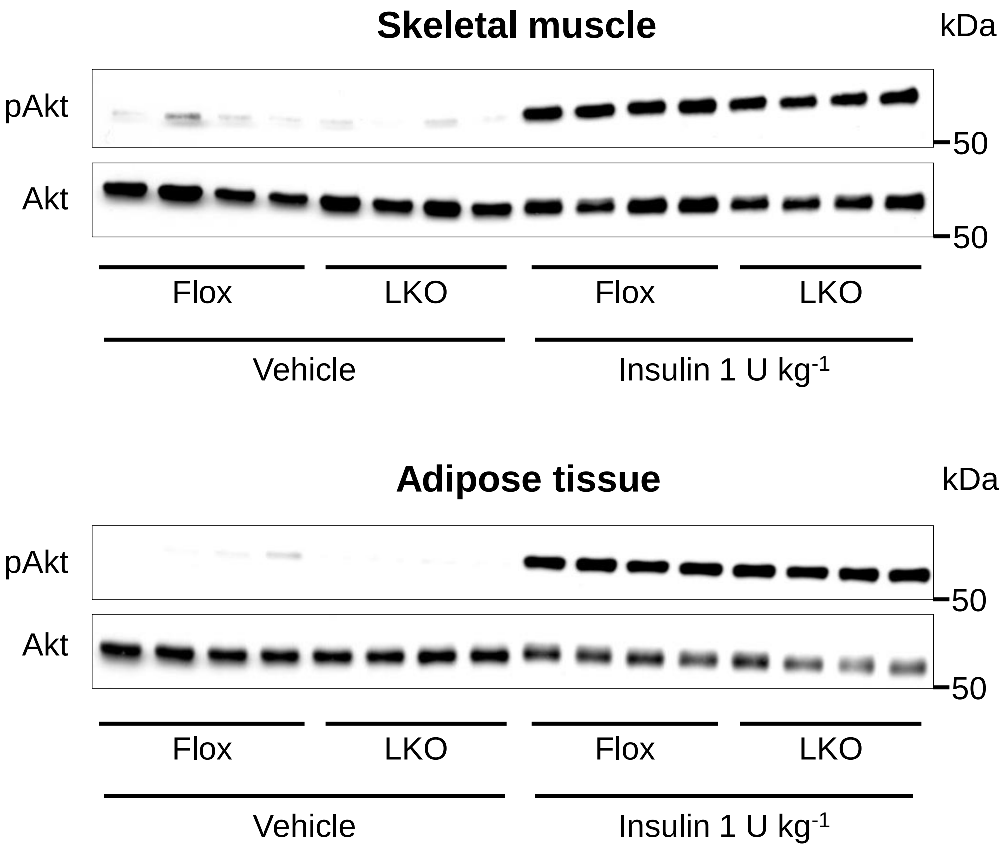

Supplementary Figure 6. Insulin-stimulated phosphorylation of Akt in skeletal muscle and adipose tissue. Western blotting for insulin (1 U kg^{-1})-stimulated phosphorylation of Akt in skeletal muscle and adipose tissue from Flox and LKO mice. Representative western blot image was shown (n=8 mice per group).

Supplementary Figure 7.

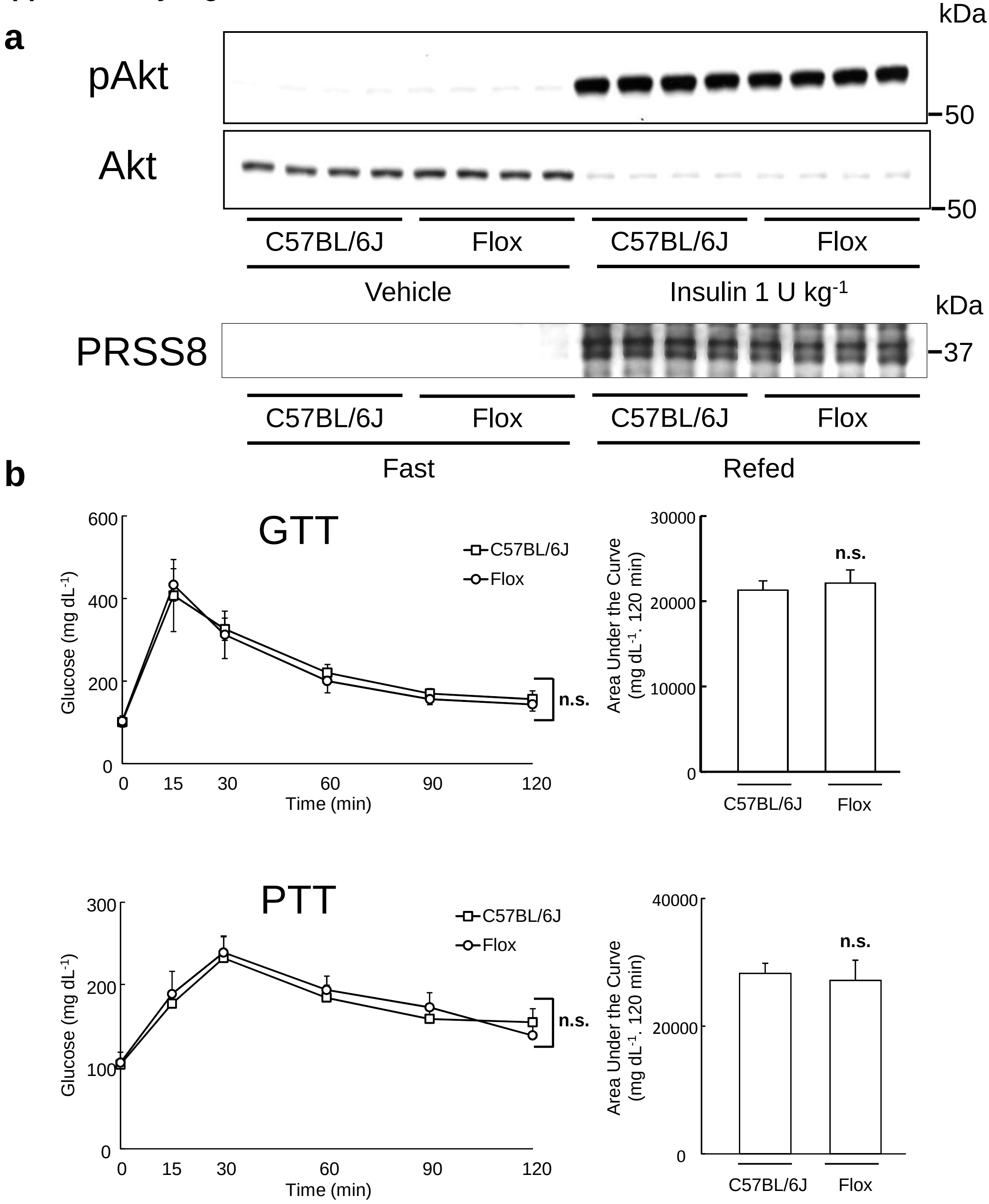

Supplementary Figure 7. Flox mice and C57BL/6J mice show similar insulin sensitivity and PRSS8 levels in the liver. (a) Western blotting for insulin-stimulated phosphorylation of Akt and levels of PRSS8 in the liver. Representative western blot image was shown (n=6 mice per group). **(b)** Blood glucose levels (two-way ANOVA) and AUC (one-way ANOVA) for GTT and PTT (n=8 mice per group). Values are shown as the mean ± s.d.

Supplementary Figure 8.

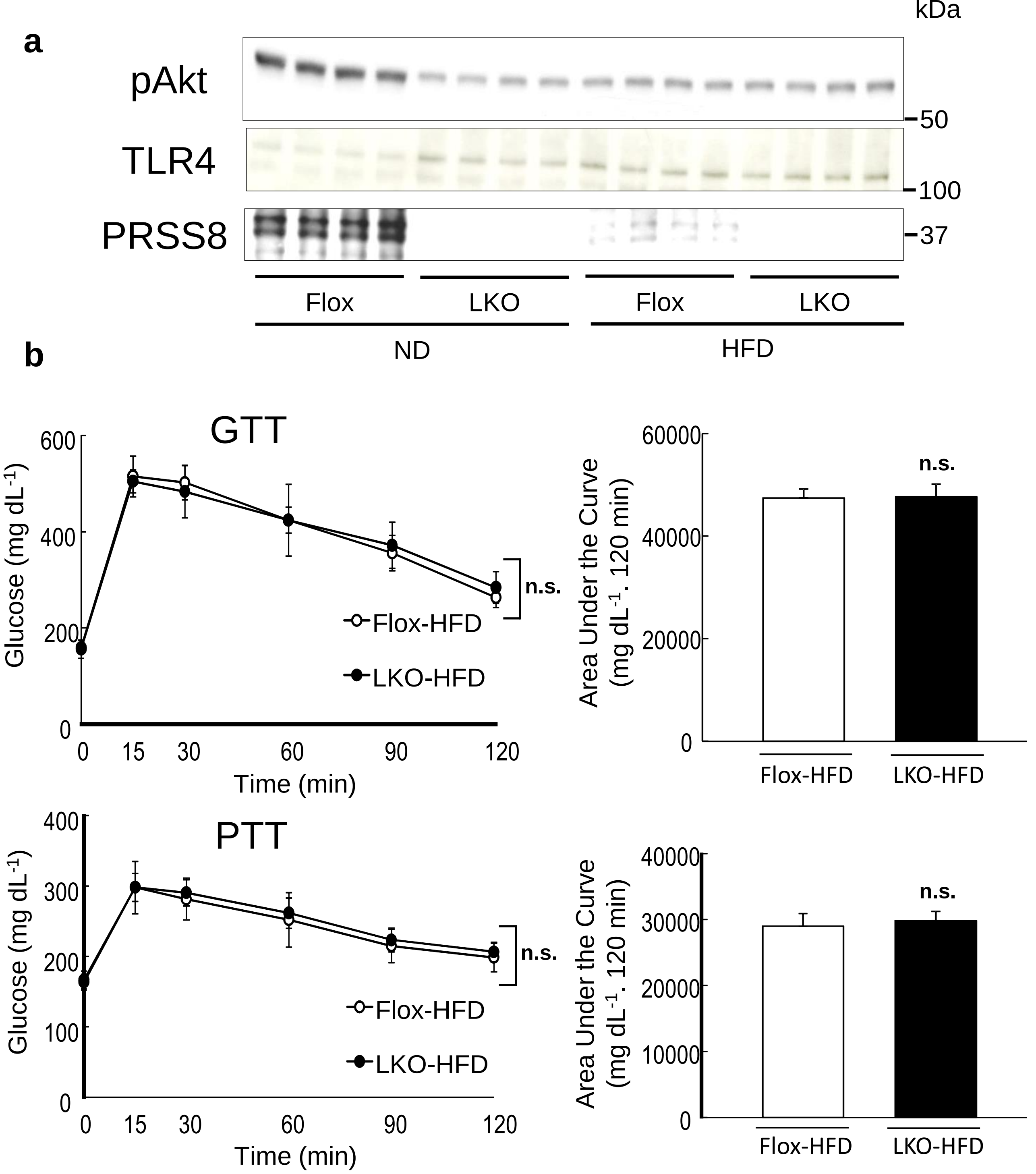

Supplementary Figure 8. Flox and LKO mice show similar insulin sensitivity and hepatic PRSS8 and TLR4 levels under HFD. (a) Western blotting for insulin-stimulated phosphorylation of Akt and levels of PRSS8 and TLR4 in the liver. Representative western blot image was shown (n=6 mice per group). **(b)** Blood glucose levels (two-way ANOVA) and AUC (one-way ANOVA) for GTT and PTT (n=8 mice per group). Values are shown as the mean ± s.d.

Supplementary Figure 9.

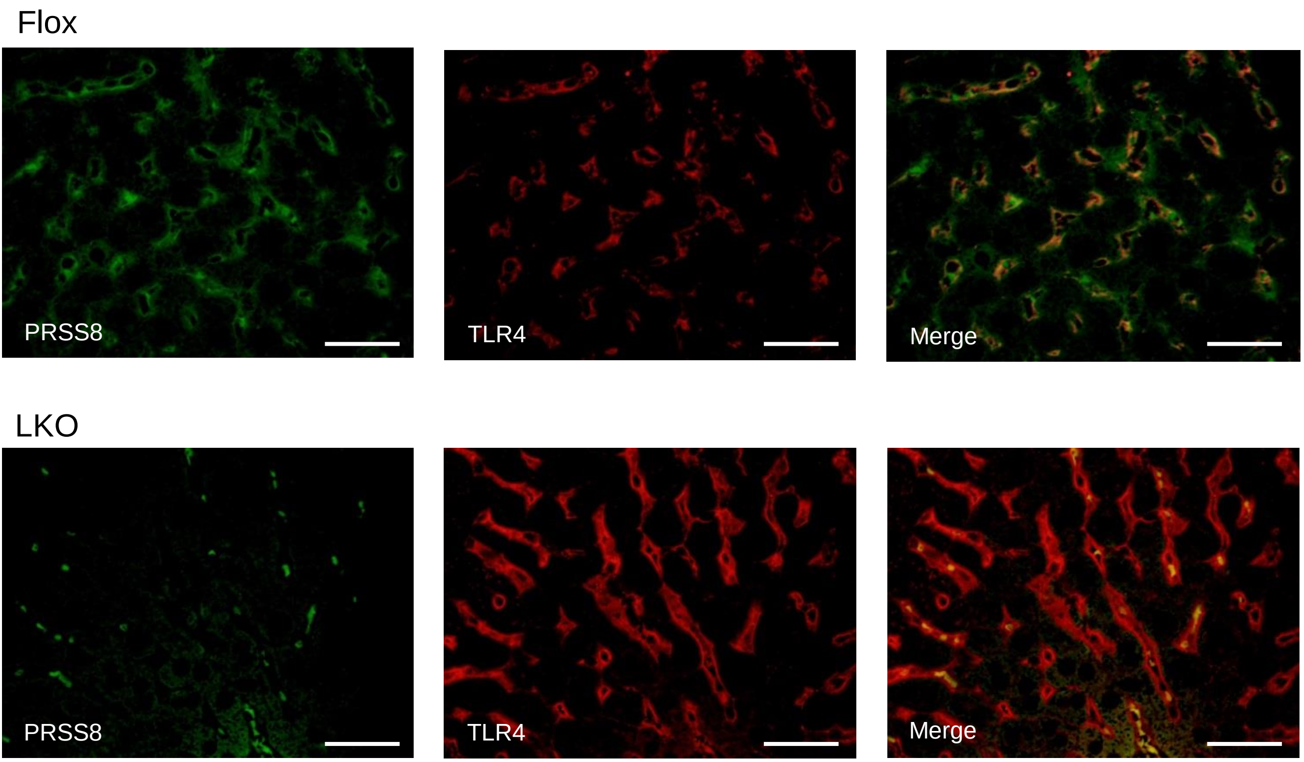

Supplementary Figure 9. Immunohistochemical analysis of PRSS8 (green) and TLR4 (red) in the livers from Flox and LKO mice.
Immunostaining was performed on PFA perfusion fixed sections. Sections were incubated with anti-PRSS8 (Proteintech) and anti-TLR4 (Abcam) antibodies, and visualized with Alexa 488 and 555. Scale bars, 40 μm.

Supplementary Figure 10.

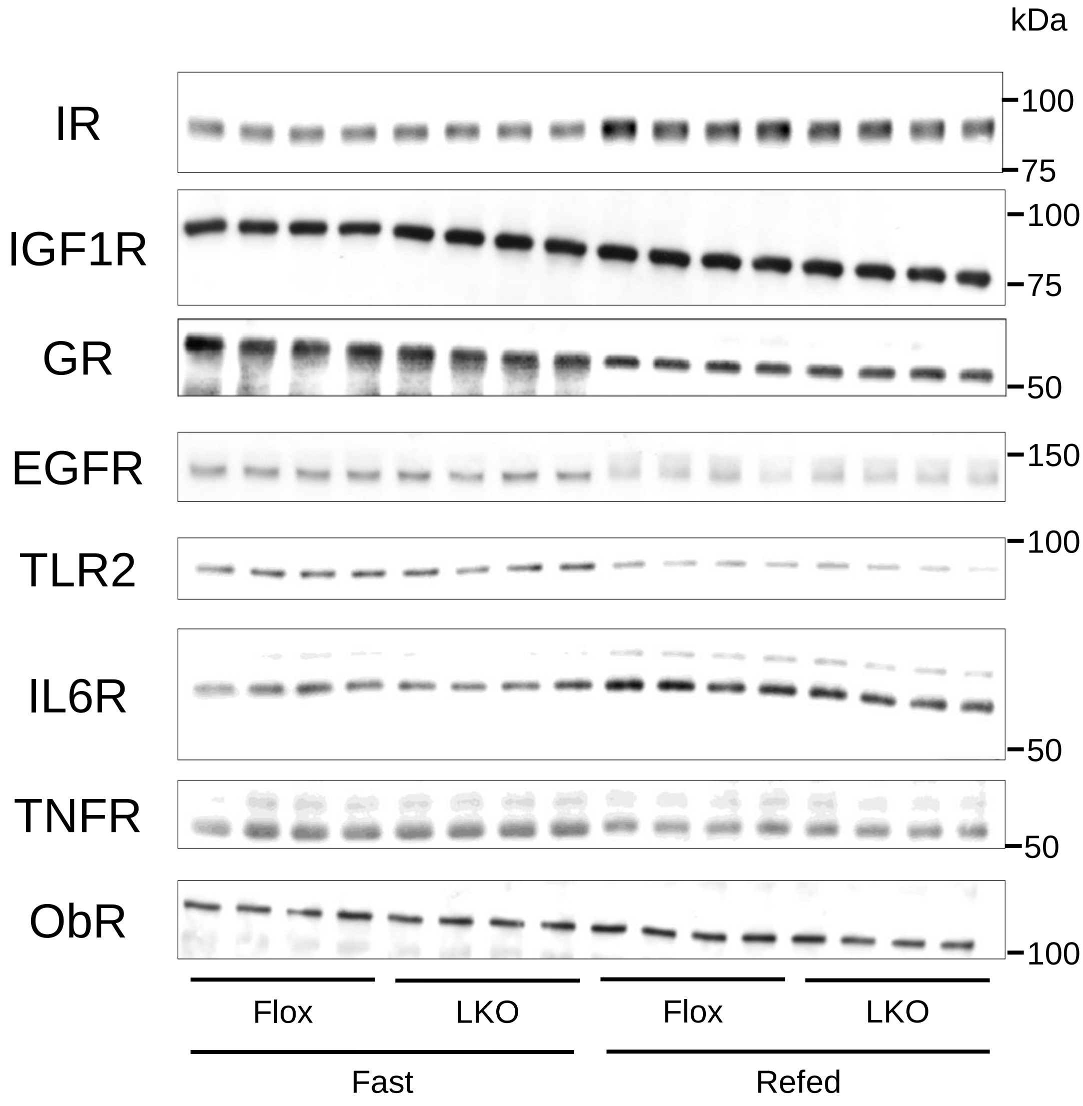

Supplementary Figure 10. Western blotting for the expression of cell surface receptors regulating inflammation or glucose metabolism in the liver from LKO and Flox mice. No significant differences in the expression of cell surface receptors regulating inflammation or glucose metabolism were observed between LKO and Flox mice under fasting or refeeding conditions. Representative western blot image was shown (n=6 mice per group). IR: Insulin receptor, IGF1R: Insulin-like growth factor 1 receptor, GR: Glucagon receptor, EGFR: Epidermal growth factor receptor, and ObR: Leptin receptor.

Supplementary Figure 11.

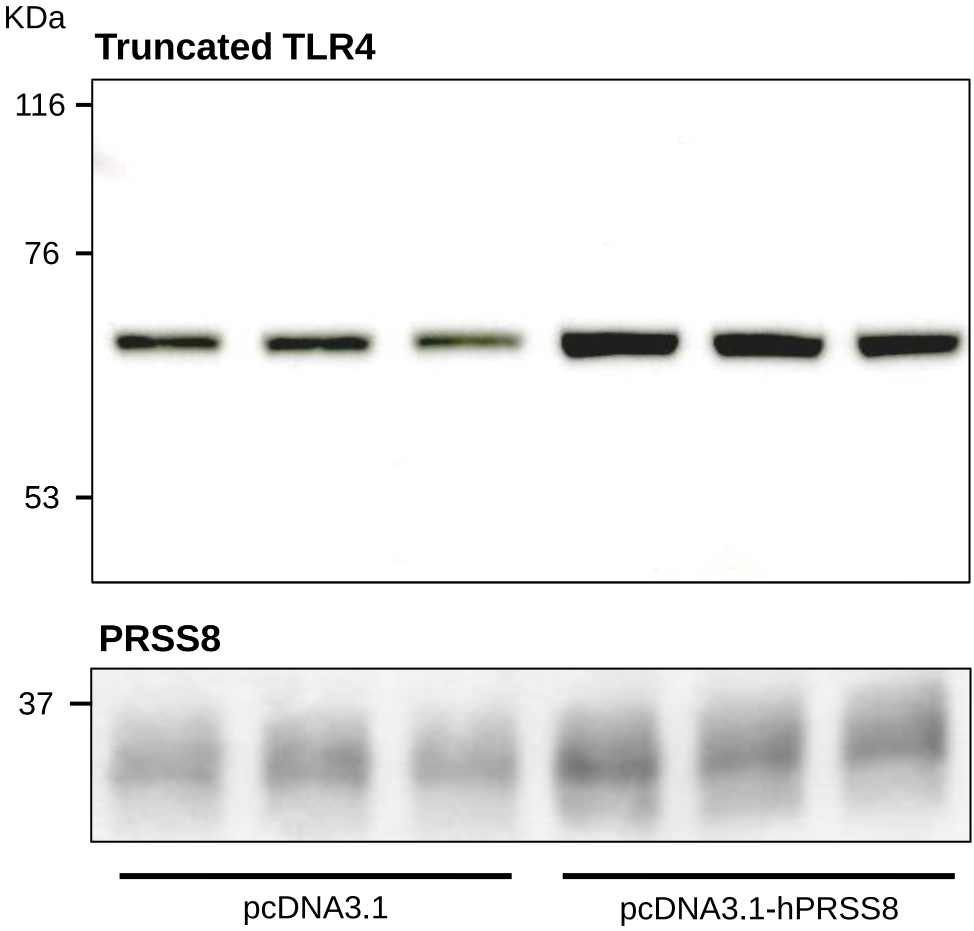

Supplementary Figure 11. Levels of TLR4 and PRSS8 in the culture media from HepG2 cells transfected with pcDNA3.1 or pcDNA3.1-hPRSS8.

HepG2 cells were transfected with pcDNA3.1 or pcDNA3.1-hPRSS8. Thirty-six hours after transfection, the culture medium was replaced with serum-free medium, and the cells were incubated for an additional 24 hr. The culture medium was collected and precipitated with TCA. The pellets were solubilized with a loading buffer and subjected to immunoblotting.

Supplementary Figure 12.

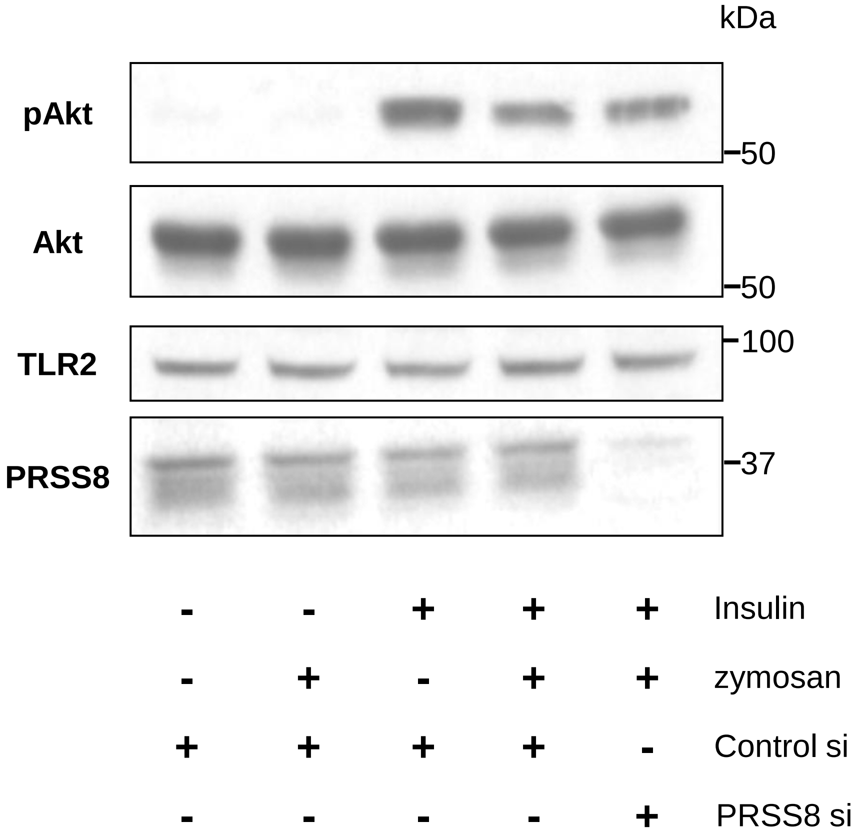

Supplementary Figure 12. TLR2 ligand zymosan did not affect the insulin-stimulated Akt phosphorylation in PRSS8-depleted HepG2 cells.
HepG2 cells transfected with Control siRNA or PRSS8 siRNA were pretreated with zymosan (100μg mL^{-1}), then insulin-stimulated phosphorylation of Akt was determined by western blotting.

Supplementary Figure 13.

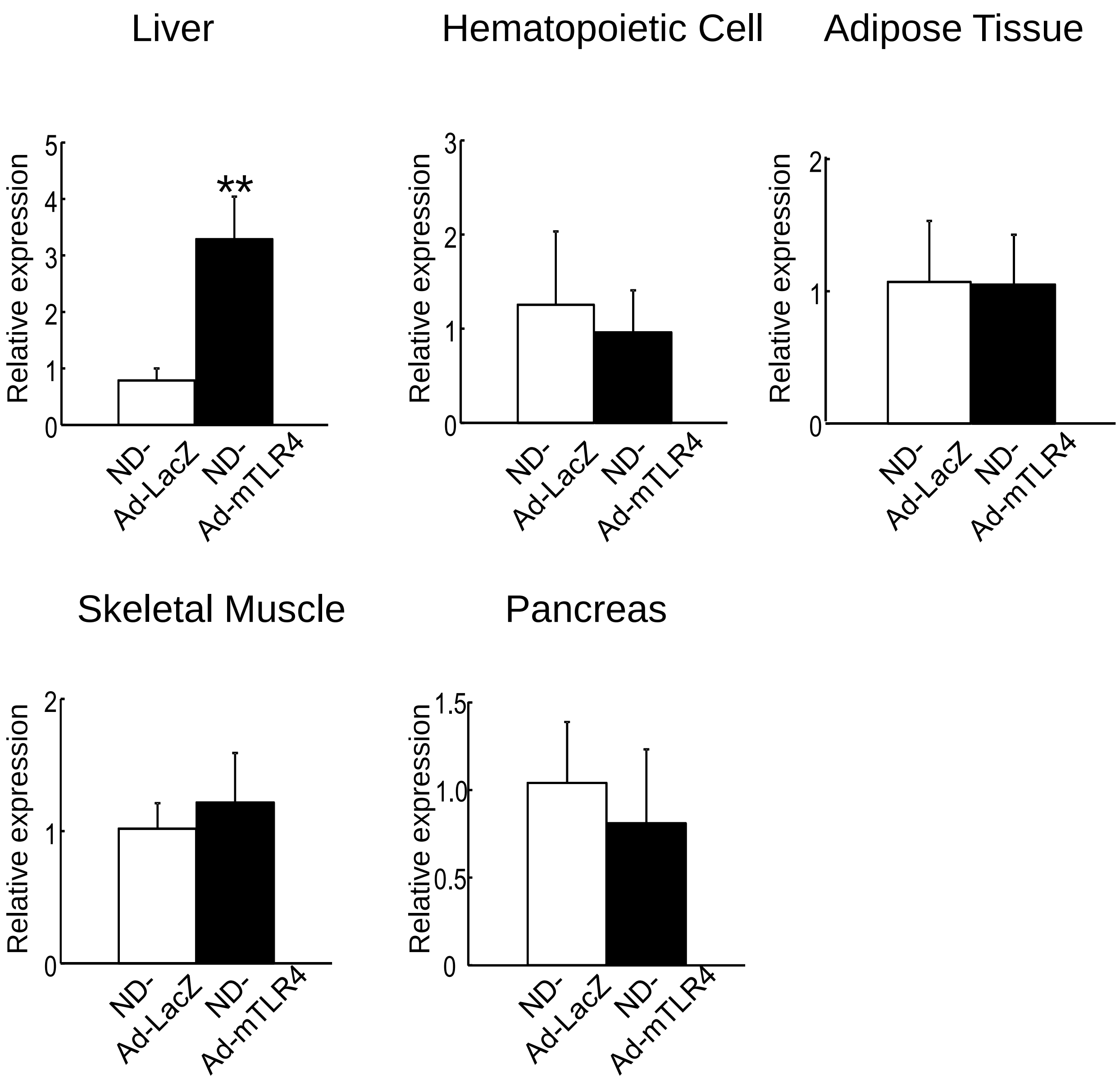

Supplementary Figure 13. Expression of TLR4 mRNA in wild type mice infected with Ad-LacZ or Ad-mTLR4 under ND (n=5-9 mice per group).
Values are shown as the mean ± s.d., ***p*<0.01 vs. ND-Ad-LacZ (one-way ANOVA).

Supplementary Figure 14.

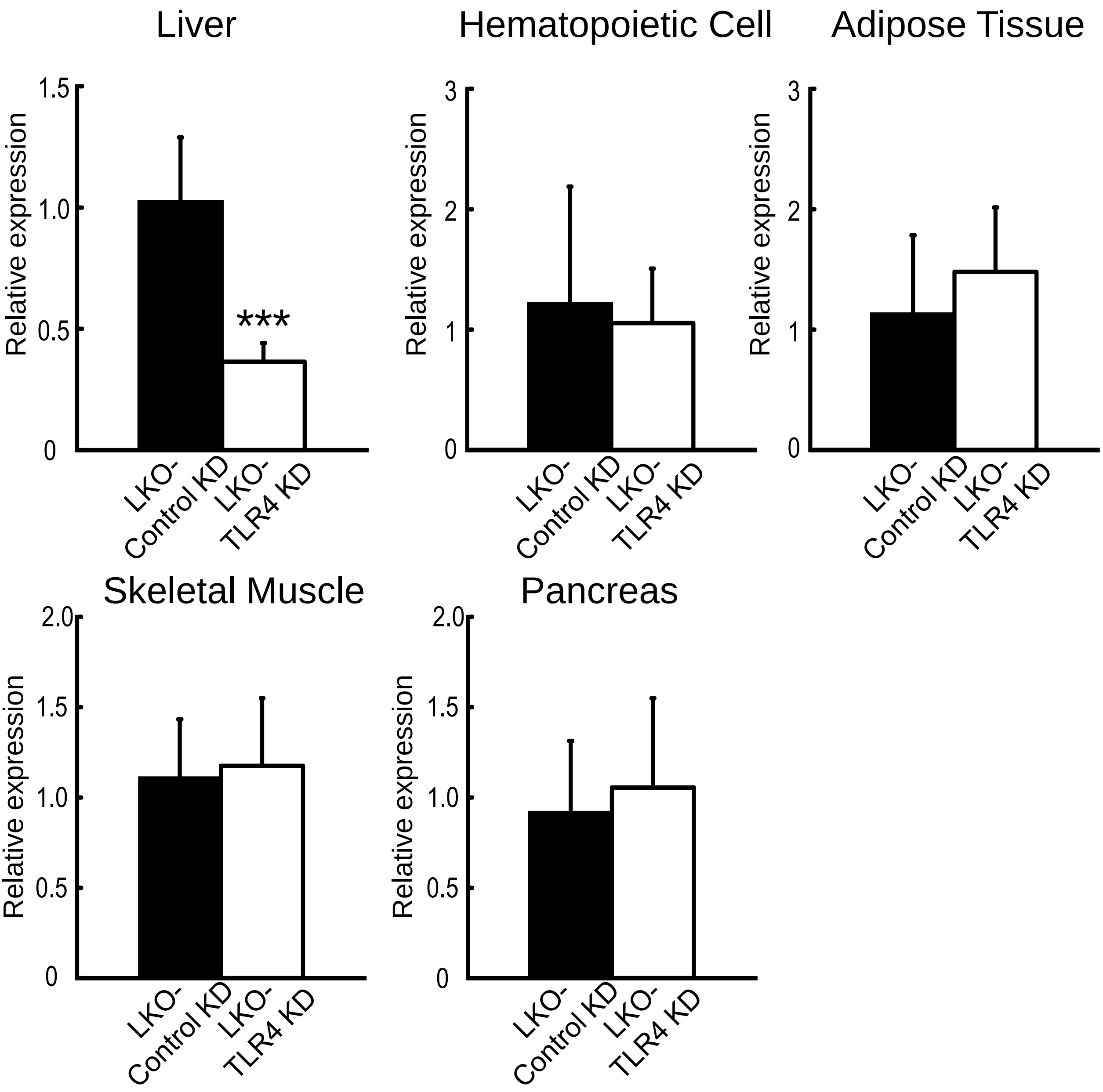

Supplementary Figure 14. Expression of TLR4 mRNA in LKO mice transfected with Control siRNA or TLR4 siRNA (n=6-9 mice per group).
Values are shown as the mean ± s.d., ****p*<0.001 vs. LKO-Control KD (one-way ANOVA).

Supplementary Figure 15.

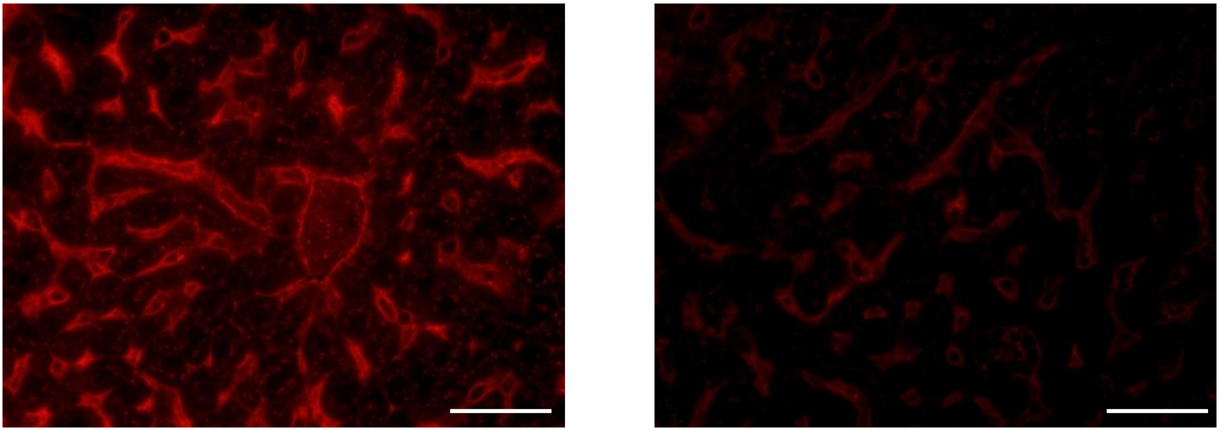

LKO-Control KD LKO-TLR4 KD

Supplementary Figure 15. Immunohistochemical analysis of TLR4 (red) in the livers from LKO mice transfected with Control siRNA or TLR4 siRNA.
Immunostaining was performed on 5-µm paraffin sections. Sections were incubated with anti-TLR4 (Abcam) antibodies and visualized with Alexa 555. Scale bars, 40 µm.

Supplementary Figure 16.

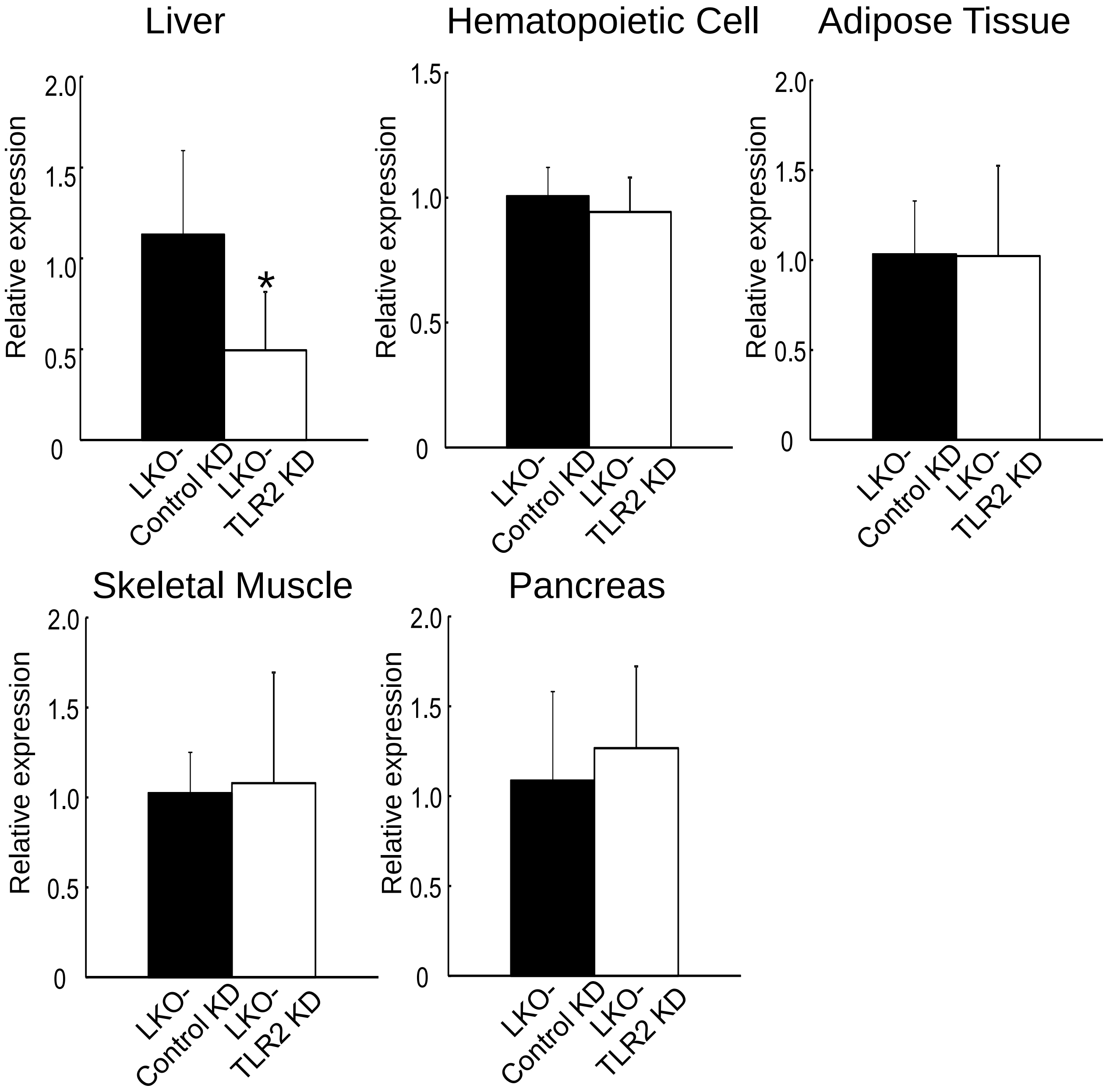

Supplementary Figure 16. Expression of TLR2 mRNA in LKO mice transfected with Control siRNA or TLR2 siRNA (n=7 mice per group).
Values are shown as the mean ± s.d., *$p<0.05$ vs. LKO-Control KD (one-way ANOVA).

Supplementary Figure 17.

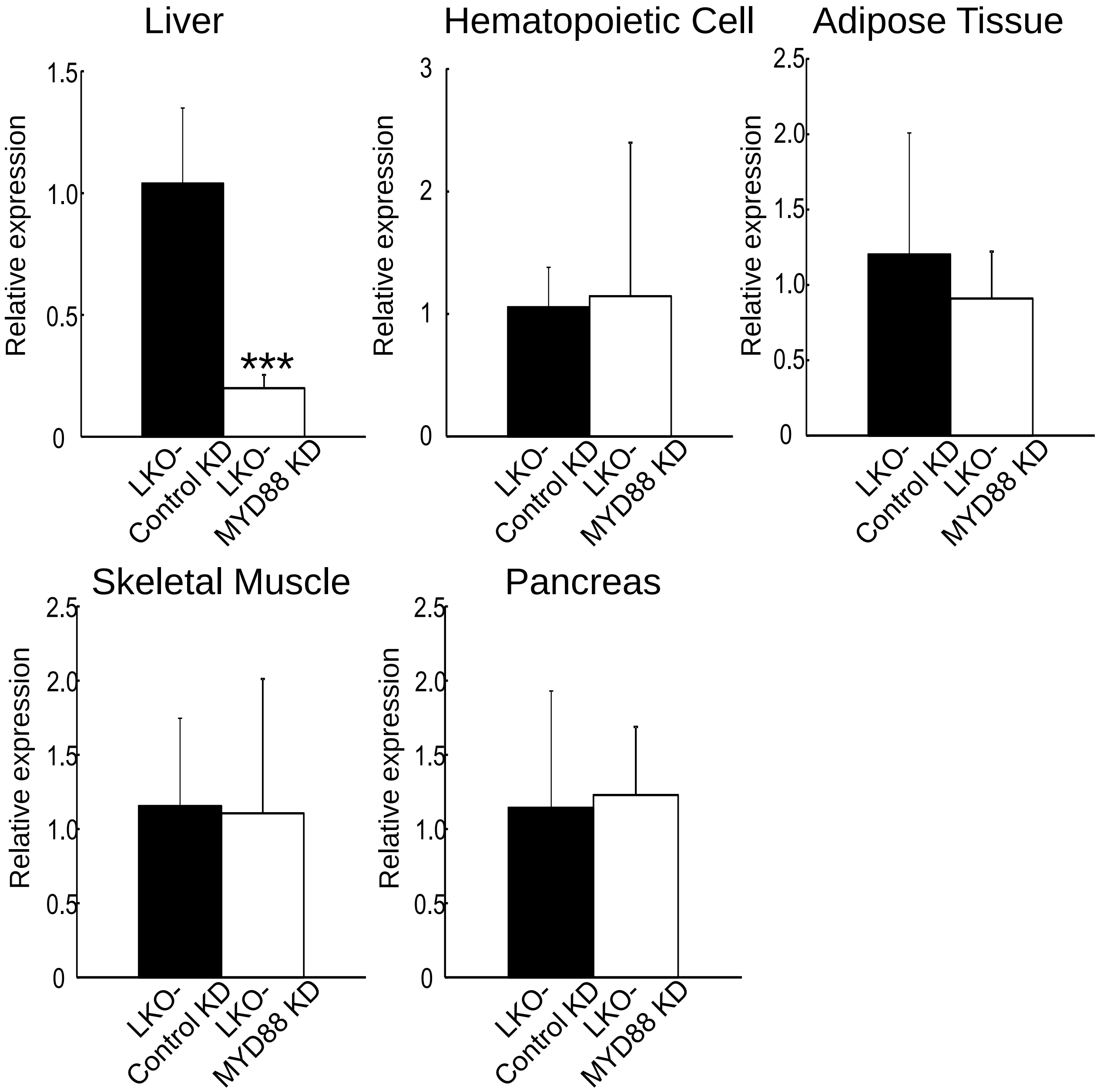

Supplementary Figure 17. Expression of MYD88 mRNA in LKO mice transfected with Control siRNA or MYD88 siRNA (n=7 mice per group).
Values are shown as the mean ± s.d., ****p*<0.001 vs. LKO-Control KD (one-way ANOVA).

Supplementary Figure 18.

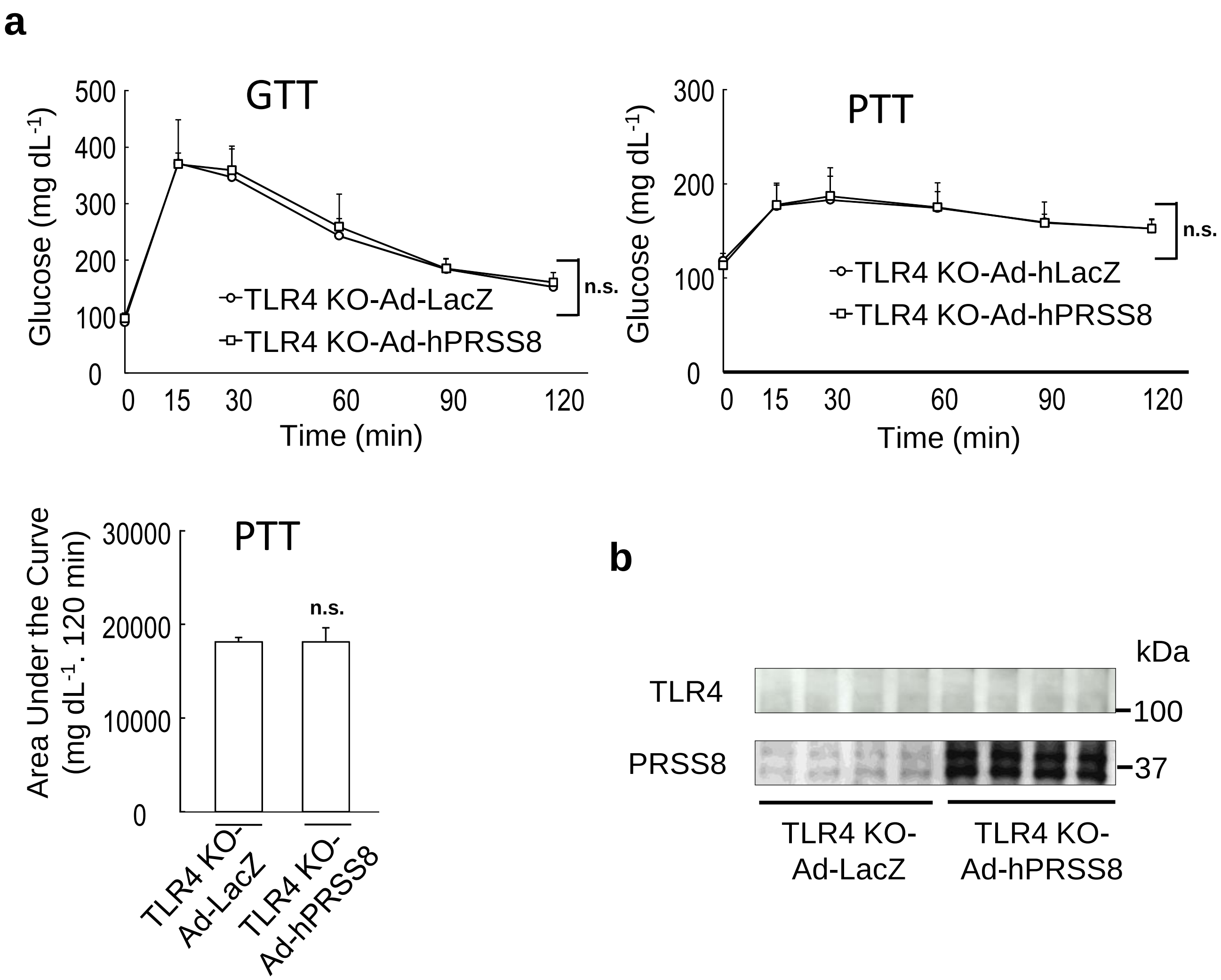

Supplementary Figure 18. Overexpression of PRSS8 does not affect insulin sensitivity in TLR4 KO.
(a) Blood glucose levels during GTT and PTT (two-way ANOVA) and AUC for PTT (one-way ANOVA). Values are shown as the mean ± s.d. (n=8 mice per group) **(b)** Western blotting for TLR4 and PRSS8 in the liver. Representative western blot image was shown (n=5 mice per group).

Supplementary Figure 19.

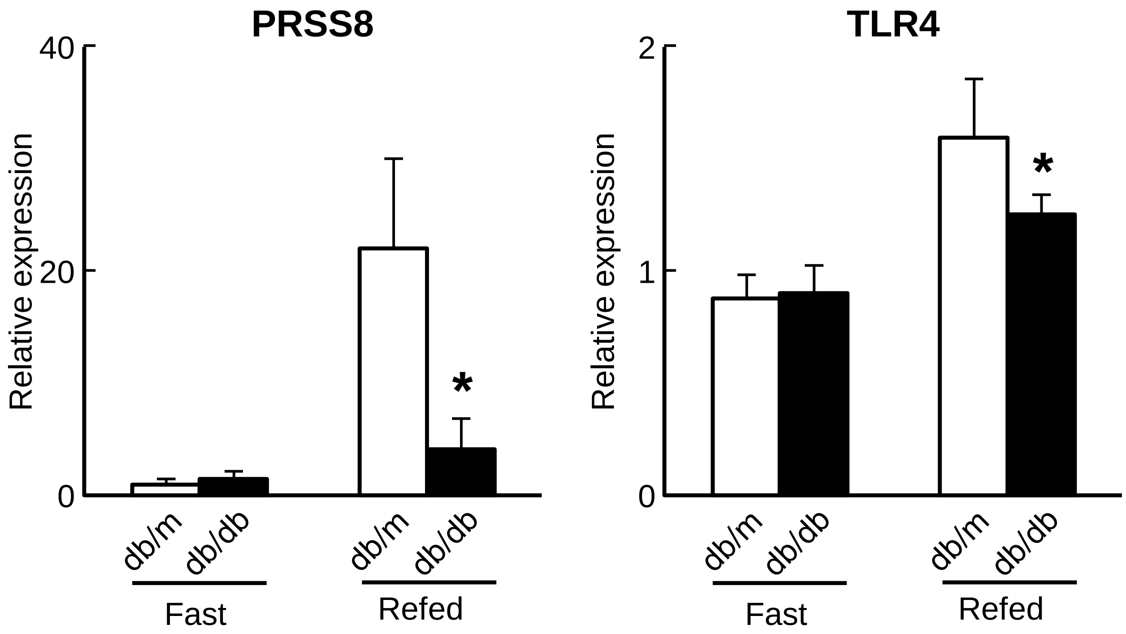

Supplementary Figure 19. mRNA expression of PRSS8 and TLR4 in the livers from *db/m* and *db/db* mice under fasting and refeeding conditions (n=6 mice per group).
*p<0.05 vs. *db/m* mice (one-way ANOVA). Values are shown as the mean ± s.d.
